# Histone Demethylase KDM7A Regulates Androgen Receptor Activity, and Its Chemical Inhibitor TC-E 5002 Overcomes Cisplatin-Resistance in Bladder Cancer Cells

**DOI:** 10.3390/ijms21165658

**Published:** 2020-08-06

**Authors:** Kyoung-Hwa Lee, Byung-Chan Kim, Seung-Hwan Jeong, Chang Wook Jeong, Ja Hyeon Ku, Hyeon Hoe Kim, Cheol Kwak

**Affiliations:** 1 Department of Urology, Seoul National University Hospital, Seoul 03080, Korea; lee12042@snu.ac.kr (K.-H.L.); dalkyal12@gmail.com (B.-C.K.); drboss@gmail.com (C.W.J.); randyku@hanmail.net (J.H.K.); hhkim@snu.ac.kr (H.H.K.); 2 Graduate School of Medical Science and Engineering, Korea Advanced Institute of Science and Technology (KAIST), Daejeon 34052, Korea; 11shjeong@gmail.com; 3 Department of Urology, Seoul National University College of Medicine, Seoul 03080, Korea

**Keywords:** bladder cancer, KDM7A, histone demethylase, TC-E 5002, androgen receptor, drug resistance

## Abstract

Histone demethylase KDM7A regulates many biological processes, including differentiation, development, and the growth of several cancer cells. Here, we have focused on the role of KDM7A in bladder cancer cells, especially under drug-resistant conditions. When the *KDM7A* gene was knocked down, bladder cancer cell lines showed impaired cell growth, increased cell death, and reduced rates of cell migration. Biochemical studies revealed that KDM7A knockdown in the bladder cancer cells repressed the activity of androgen receptor (AR) through epigenetic regulation. When we developed a cisplatin-resistant bladder cancer cell line, we found that AR expression was highly elevated. Upon treatment with TC-E 5002, a chemical inhibitor of KDM7A, the cisplatin-resistant bladder cancer cells, showed decreased cell proliferation. In the mouse xenograft model, KDM7A knockdown or treatment with its inhibitor reduced the growth of the bladder tumor. We also observed the upregulation of KDM7A expression in patients with bladder cancer. The findings suggest that histone demethylase KDM7A mediates the growth of bladder cancer. Moreover, our findings highlight the therapeutic potential of the KMD7A inhibitor, TC-E 5002, in patients with cisplatin-resistant bladder cancer.

## 1. Introduction

Bladder cancer (BCa) is one of the most common cancers in men, resulting in a reported 8470 new cases and over 17,670 deaths in the United States in 2019 [1]. BCa has a high prevalence of recurrence and metastatic spread, and the 5-year survival rate has remained relatively low, despite the advances in various surgical and chemotherapeutic treatment options [2]. The incidence of BCa is three times higher in men than women, and is the 4th and 11th most common cancer in men and women, respectively [3]. The variation in prevalence depending on gender has prompted a number of studies into the role of sex hormone receptors in BCa. Notably, emerging evidence has supported a role for the androgen receptor (AR) in BCa [4]. AR is a well-known transcription factor that responds to male sex hormones, and controls prostate cancer development and metastasis [5,6]. Recently, many studies have highlighted its role in other cancer types, including colon [7], breast [8], stomach [9], and bladder cancer [10,11,12,13]. A reduced incidence of N-butyl-N-(4-hydroxybutyl)-nitrosamine (BBN)-induced BCa has been reported in both full-body [14] as well as urothelial-specific [15] AR knock-out mice models. Recent preclinical studies have suggested that the androgen-mediated AR signaling promotes bladder cancer progression, and blocking this signaling with enzalutamide can strongly impair bladder cancer cell growth [16,17,18]. Another recent study has shed light on the role of the AR in cisplatin-resistant bladder cancer [19]. It is also reported that the anti-androgenic drug hydroxyflutamide increased cisplatin sensitivity in cisplatin-resistant bladder cancer cell line T24.

Since AR is a transcription factor, controlling its transcriptional activity can be a major target for anti-cancer drug development. Among the mechanisms known to regulate transcriptional activity, epigenetic regulation, including DNA methylation and histone modification, plays a key role in cancer development [20,21]. Specifically, histone methylation on lysine residues of histone H3 or H4 are known to modify transcriptional activity, depending on the residues modified. Methylation on lysine 4 (H3K4) or 36 (H3K36) is usually associated with transcriptional activation, while H3K9 and H3K27 methylation are frequently linked with gene silencing, and are hallmarks of chromatin condensation [22,23]. Each methylase and demethylase has specific target promoters; therefore, inhibitors can be used for targeting the regulation of a specific gene. Since the epigenetic modifications play important roles in cancer formation, malignancy, and metastasis, targeting the epigenetic enzymes would be a promising approach in cancer therapy. Indeed, epigenetic processes which control AR activity have been reported to play a role in prostate cancer development [24,25]. For the regulation of histone methylation on its target promoters, AR is known to interact with several enzymes, including LSD1, KDM4B, KDM5B, EZH2, SMYD3, PRMT5, and KDM7A [26,27,28,29,30,31,32].

Among the histone modifying enzymes, the enzyme KDM7A belongs to a family of plant homeodomain finger proteins, that contain a plant homeodomain (PHD) and a JmjC domain. The methyl groups of the lysine at positions 9 and 27 in histone 3 can be removed by the JmjC domain-containing family of proteins [33]. Since the methylation of H3K9 and H3K27 represent the repression of gene expression, their demethylation activates target gene transcription. Previous studies have shown that KDM7A regulates bone development, adipogenesis, inflammation, as well as the development of various types of cancers [34,35,36]. A recently published paper from our research group described the H3K27 demethylase activity of KDM7A on the response elements of AR target genes in prostate cancer [32]. The study presents a detailed account of the physical interaction of KDM7A with AR, using the immune-precipitation method. Furthermore, we found that KDM7A directly binds to the androgen response element (ARE) sequences of AR target genes, including KLK3, KLK2, and TMPRSS2. An increase in the histone H3K27 di-methylation of those ARE sequences and a decrease in the AR activity was also observed in KDM7A knockdown prostate cells. The existence of a KDM7A chemical inhibitor further highlighted the value of this data, owing to its clinical application as an anti-cancer drug.

In the present study, we have further investigated the role of KDM7A in the epigenetic regulation of AR in BCa, with a focus on the regulation of AR activity in the cisplatin-resistant bladder cancer cells.

## 2. Results

### 2.1. KDM7A Regulates AR Transcription Activity in Bladder Cancer Cells

**Figure 1:**
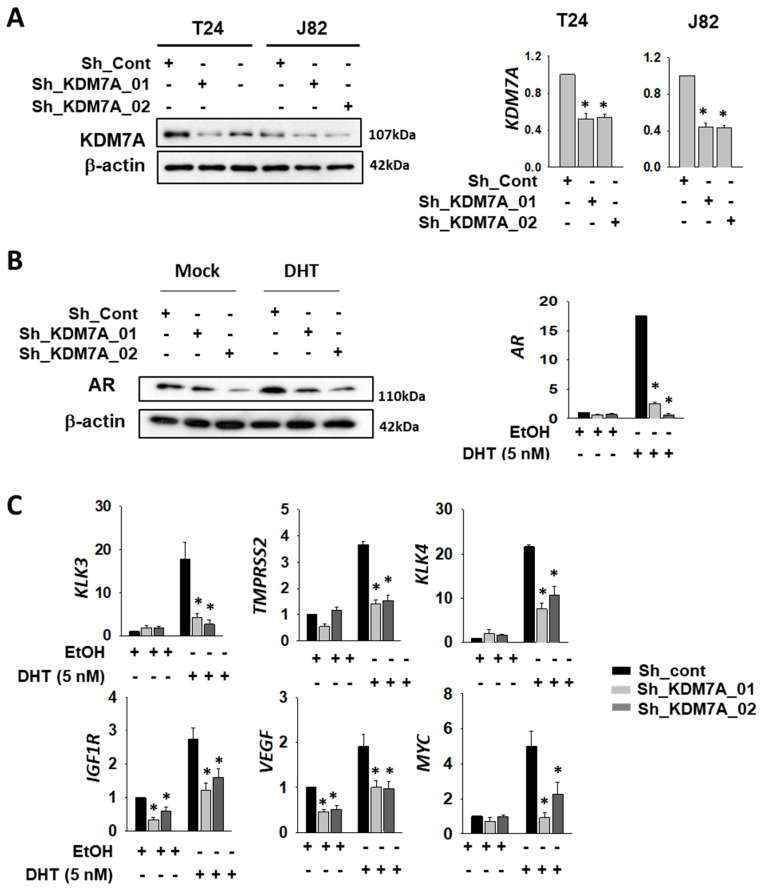
Histone demethylase KDM7A is required for AR activity in bladder cancer cells. (**A**) The efficiency of KDM7A knock-down was measured by comparing protein levels of KDM7A in the indicated cell lines, expressing control or two different shRNAs. Whole-cell lysates were analyzed with the indicated antibodies (left). The mRNA levels of KDM7A were measured by RT-qPCR method in the KDM7A knock-down cell lines (right graphs). Bars represent the means ± SD of three independent experiments, and * denotes *p* < 0.05 (student *t*-test) versus the control shRNA (sh-cont) group. (**B**) The AR protein levels in KDM7A knock-down T24 cells treated with dihydrotestosterone (DHT) were analyzed with indicated antibodies (left). The mRNA levels of AR in T24 cells after DHT induction were measured by RT-qPCR (right graph). Bars represent the means ± SD of three independent experiments, and * denotes *p* < 0.05 (student *t*-test) versus the control shRNA (sh-cont) group. (**C**) The mRNA levels of AR downstream genes were measured by RT-qPCR method in KDM7A knock-down T24 cell lines. Bars represent mean ± SD of three independent experiments. * *p* < 0.05 (Student’s *t*-test), versus the control shRNA (sh-cont) group.

In order to investigate the possible role of KDM7A demethylase in the functioning of AR in bladder cancer cells, we first compared the AR expression levels in various bladder cancer cell lines, including 253J, RT4, T24, and J82. As expected, the levels of AR mRNA and protein in bladder cancer cells were quite low compared to that in LNCaP prostate cancer cells (Supplemental Figure S1A,B). Nonetheless, we were able to detect AR mRNA and protein in the bladder cancer cells that we tested, with the levels found to be comparable among them. Since a previous study has reported the development of cisplatin-resistant T24 cells [19], we selected this cell line for our experiments. We also included another bladder cancer cell line, J82, in order to demonstrate data reproducibility. To analyze the function of KDM7A histone demethylase in bladder cancer cells, we produced KDM7A knock-down bladder cell lines (T24 and J82), using a lenti-viral shRNA expression system. After antibiotics selection, efficient knock-down of the gene expression was confirmed by Western blotting and reverse transcriptase quantitative PCR (RT-qPCR) (Figure 1A). Since KDM7A is known to regulate AR activity in prostate cancer cells [32], we speculated that it may control AR activity as an epigenetic regulator in bladder cancer cells. Our studies showed that the levels of both AR mRNA and protein were regulated by KDM7A, before and after dihydrotestosterone (DHT) induction (Figure 1B, Supplemental Figure S2), even though we had expected changes only in the protein activity. A possible explanation could be the autoregulatory effect of AR on its own promoter [37]. In order to measure AR activity in KDM7A knock-down cells, the expression of previously reported downstream target genes of AR was screened using RT-qPCR [38,39,40,41,42,43]. The relative mRNA levels of these genes, compared with those of LNCaP, are listed in Supplemental Figure S1C. The PCR signals of six genes (*KLK3, TMPRSS2, KLK4, IGF1R, VEGF,* and *MYC*) were successfully amplified in bladder cancer cell lines. The mRNA levels of these AR target genes were elevated after DHT treatment, and this induction was reduced in KDM7A knock-down stable cells (Figure 1C).

### 2.2. KDM7A Regulates AR Transcription Activity via Epigenetic Regulation of AR Target Gene Promoters

**Figure 2:**
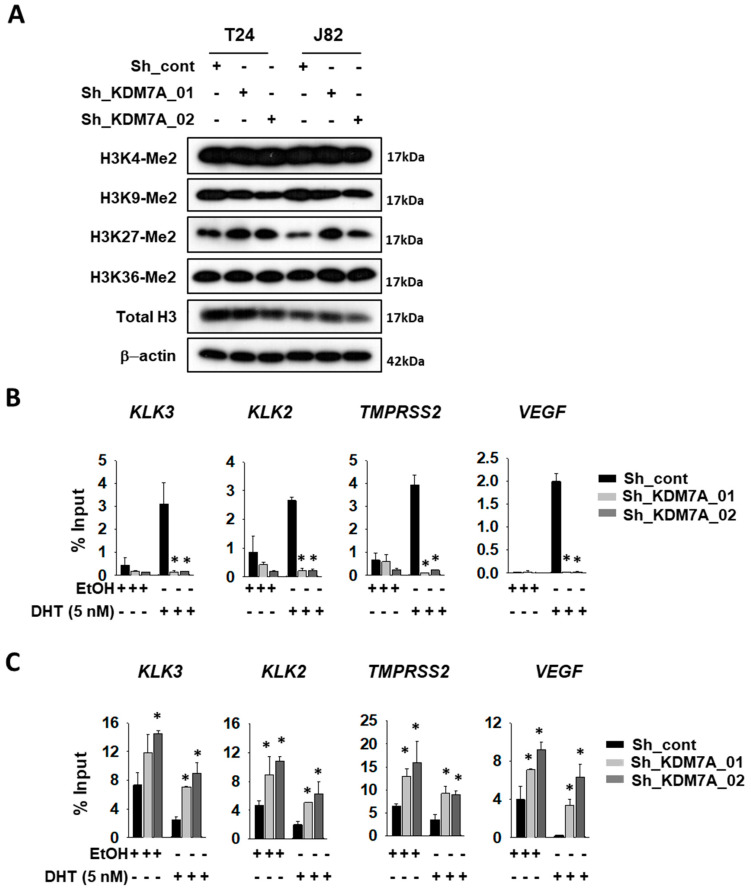
KDM7A directly binds on androgen receptor (AR) downstream gene promoters, and regulates H3K27 methylation. (**A**) Histone methylation status in KDM7A knock-down cells was analyzed with the indicated antibodies. T24 cells expressing control or KMD7A shRNA were treated with 5 nM DHT for 1 day, and the sonicated chromatins were immune-precipitated with anti-AR (**B**) or anti-H3K27 di-methyl (**C**) antibody. Immunoprecipitated DNAs were subjected to qPCR with the indicated gene promoter sequence primers. Bars represent means ± SD of three independent experiments. * *p* < 0.05 (Student’s *t*-test), versus the control shRNA (sh-cont) group.

Next, we analyzed the histone methylation status in KDM7A knock-down cells. The cell extracts from each of the cell lines were analyzed with specific antibodies for diverse histone methylation sites (Figure 2A). Among the sites tested, only H3K27 di-methylated lysine was elevated in KDM7A knock-down cells. The AR activity can be measured as the extent of binding of AR onto target promoters. We, therefore, analyzed the binding efficiency of AR to its target promoters, using the chromatin immunoprecipitation (ChIP) experiment. The T24 bladder cancer cells expressing control or KDM7A shRNA were treated with DHT, sonicated, and the chromatin was precipitated with AR antibody. The promoter binding by AR on the indicated genes was detected with IP PCR. The precise location of the PCR primers for each of the gene promoters is described in Supplemental Figure S3. Induction with DHT was shown to increase the binding of AR to these promoters, while this induction was abolished in the KDM7A knock-down cells (Figure 2B). We next attempted to investigate methylation statuses on AR responsive promoters in the KDM7A knock-down cells. Based on the changes in methylation (Figure 2A), we used H3K27 di-methyl-specific histone antibodies for immunoprecipitations, and H3K27 methylation was found to be increased in KDM7A knock-down bladder cells, before and after DHT induction (Figure 2C). Our data confirmed the molecular function of KDM7A on AR transcription factor activity in bladder cancer cells.

### 2.3. KDM7A is Required for Bladder Cancer Cell Growth and Apoptosis Inhibition

**Figure 3:**
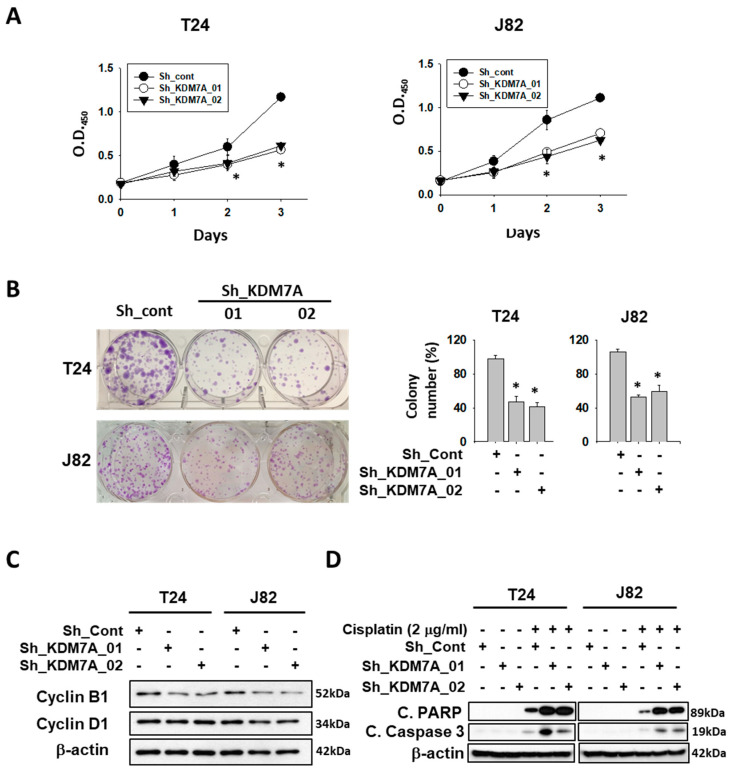
Histone demethylase KDM7A is required for the cell proliferation and apoptosis inhibition in bladder cancer cell lines. (**A**) The time dependent viability changes of control shRNA and KDM7A shRNA expressing bladder cancer cell lines were measured using EZ-Cytox solution. Bars represent means ± SD of three independent experiments. * *p* < 0.05 (Student’s *t*-test) versus the control shRNA (sh-cont) group. (**B**) Crystal violet staining for colonies from the same number of indicated shRNA-expressing stable cells. The average number of colonies is shown in the right panel. Bars represent means ± SD of three independent experiments. * *p* < 0.05 (Student’s *t*-test) versus the sh-cont group. (**C**) The levels of cell cycle-related proteins were measured from the whole cell extracts of control and KDM7A knock-down cells. (**D**) The apoptotic proteins were detected in the cisplatin-treated KDM7A knock-down bladder cells. Whole-cell lysates were analyzed with the indicated antibodies.

Since AR is known to be essential for cell growth in many cancers, we treated AR siRNA or enzalutamide, to confirm the growth inhibition effect on bladder cancer cell growth (Supplemental Figure S4). Next, we measured cell proliferation in both control and KDM7A knock-down cells. Although the morphology of KDM7A knock-down cells was not different from control cells (Supplemental Figure S5), the rate of cell proliferation in KDM7A knock-down bladder cancer cells was reduced compared to that of control shRNA-expressing cells (Figure 3A). When we seeded the same number of cells in cell culture dishes, knock-down cells showed a reduction in colony numbers and size compared to control cells (Figure 3B). We next observed the expression levels of cell cycle proteins in KDM7A knock-down cells (Figure 3C). Previous studies have reported that AR regulates cell cycle by controlling cyclin D1 [44], and cyclin B1 is a direct target of AR [45]. We observed a decrease in the cyclin B1 protein levels in KDM7A knock-down bladder cancer cells, which is consistent with the cell proliferation rate difference data in Figure 3A,B. It was observed that the decrease in cyclin D1 is not as obvious as that in cyclin B1 in our experimental conditions. Since AR is known to inhibit apoptosis induced by cytotoxic stimuli [46], we measured the levels of apoptotic proteins in KDM7A knock-down cells treated with the anti-cancer drug cisplatin (Figure 3D). We observed an increase in the PARP and caspase 3 cleavage products compared to control shRNA-expressing cells. These results strongly suggest that KDM7A regulates the rate of cell proliferation and drug-induced apoptosis in bladder cancer cells.

### 2.4. KDM7A Facilitates Migration and Invasion of Bladder Cancer Cells

**Figure 4:**
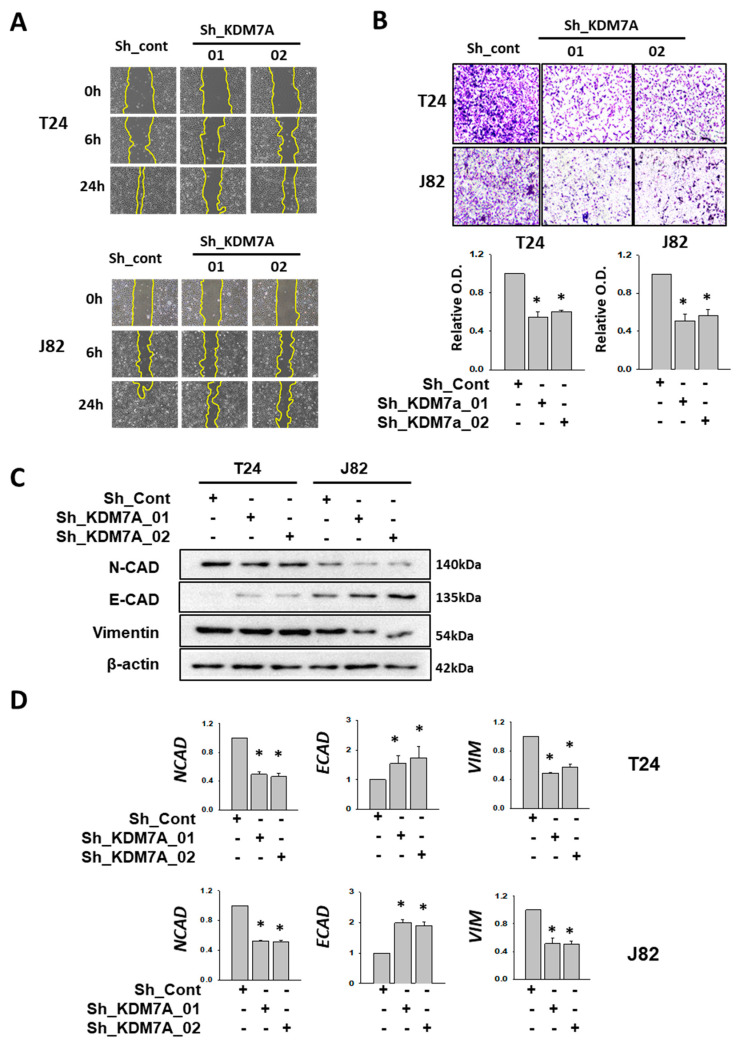
KDM7A knock-down reduced cell mobility and EMT-related gene expressions in bladder cancer cells. (**A**) Scratch-wounding cell migration assay of the control and KDM7A shRNA-expressing cells was performed for the indicated time. The wound margin is marked with a yellow line. (**B**) The Transwell assay of the same number of control and KDM7A shRNA-expressing cells. At 48 h after plating, cells that had migrated to the underside of the filters were fixed and stained with crystal violet. Photographs were taken and the relative cell migration was determined by measuring OD_495_ after extraction. Bars represent means ± SD of three independent experiments. * *p* < 0.05 (Student’s *t*-test) versus the control shRNA (sh-cont) group. (**C**) The protein levels of indicated EMT markers were measured from the whole cell extracts of control or KDM7A knock-down cells. (**D**) The mRNA levels of the indicated EMT marker genes were measured from cell lines with control or KDM7A shRNA expression. Bars represent means ± SD of three independent experiments * *p* < 0.05 (Student’s *t*-test) versus the sh-cont group.

One of the major functions of AR in cancer progression is to facilitate cell migration and metastasis. It was observed that AR inhibition by enzalutamide affected the migration of bladder cancer cells (Supplemental Figure S6). To verify the effect of KDM7A in bladder cancer cell migration and metastasis, we measured migration and invasion in KDM7A knock-down T24 and J82 bladder cancer cells. One day after scratch, KDM7A knock-down cells showed decreased mobility when compared to control cells (Figure 4A). We demarcated the wound margin with a yellow line for better visualization, and added the original pictures and quantification of remaining scratched areas after the indicated times in Supplemental Figure S7. The cell invasion assay using Matrigel Transwell showed the decreased invasion of KDM7A knock-down cells compared to control cells (Figure 4B). To elucidate the molecular mechanism of this decrease in cell migration, we measured the expression of several epithelial-mesenchymal transition (EMT) markers in KDM7A knock-down cells. Although a decrease in the protein levels of N-cadherin and vimentin, the epithelial markers, was observed only in J82 cells (Figure 4C, Supplemental Figure S8), the mRNA expression levels of them were reduced in both T24 and J82 cells (Figure 4D). The protein and mRNA levels of mesenchymal marker E-cadherin were elevated in KDM7A knock-down cells in both T24 and J82 cells (Figure 4C,D, Supplemental Figure S8). The findings suggest that KDM7A is required for cell migration and EMT transition in bladder cancer cells.

### 2.5. Enzalutamide and a KDM7A Inhibitor Decrease the Proliferation of Cisplatin-resistant Bladder Cancer Cells

**Figure 5:**
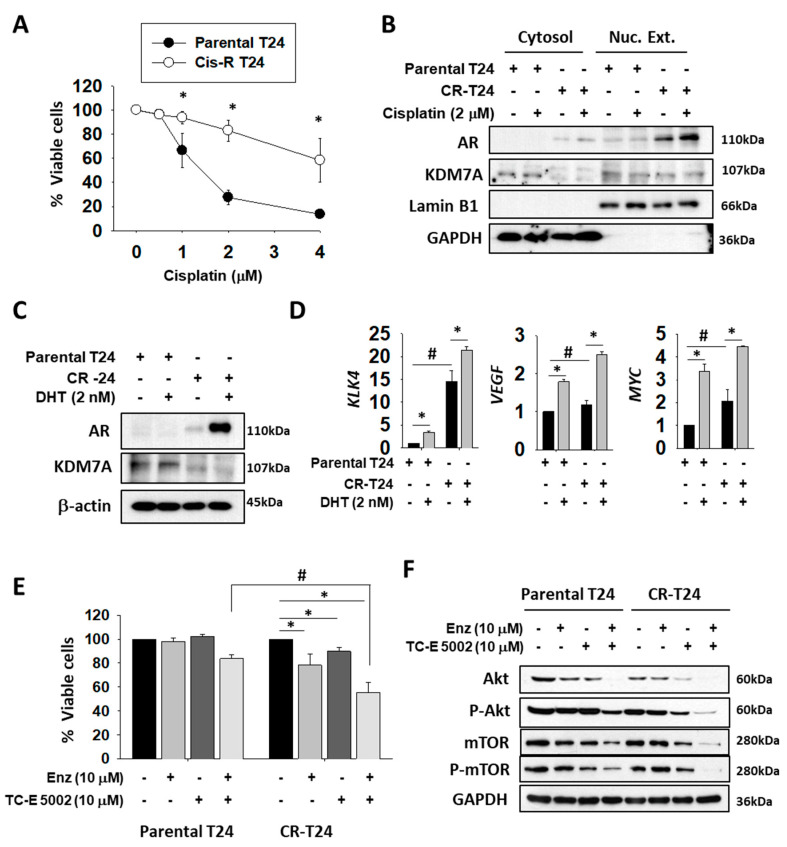
Nuclear localization and activity of AR were elevated in cisplatin-resistant T24 (CR-T24) cells, and treatment with enzalutamide and KDM7A inhibitor reduced the growth of CR-T24 bladder cancer cells. (**A**) Viability of parental and CR-T24 cells after treatment with the indicated concentrations of cisplatin for 3 days. Bars represent means ± SD of three independent experiments. * *p* < 0.05 (Student’s *t*-test) versus parental cells. (**B**) Western blots of the indicated proteins from parental and CR-T24 cells. The cytosolic and nuclear fractions from each cell line were extracted and blotted with the indicated antibodies. Lamin B1 immunostaining served as the nuclear protein loading control and GAPDH immunostaining as cytoplasmic control. (**C**) Western blots of AR and KDM7A proteins from parental and CR-24 cells, before and after 5α-dihydrotestosterone (DHT) treatment. (**D**) mRNA levels of the indicated genes were measured from parental and CR-T24 cells. Bars represent means ± SD of three independent experiments. * *p* < 0.05 (Student’s *t*-test), versus the mock-treated group. # *p* < 0.05 (Student’s t-test) versus parental T24 cells. (**E**) Cell viability changes after 3 days of treatment with the indicated drugs on parental or CR-T24 cells. Bars represent means ± SD of three independent experiments. * *p* < 0.05 (Student’s *t*-test) versus the mock-treated group. # *p* < 0.05 (Student’s *t*-test) versus parental T24 cells. (**F**) Levels of the indicated proteins were measured from whole cell extracts of parental and CR-T24 cells, treated with either enzalutamide and/or TC-E 5002 for 2 days.

The effect of AR induction on the cisplatin resistance process of bladder cancer has been previously reported [19]. Therefore, we wanted to explore the possibility of AR regulation as a target for overcoming the drug resistance of bladder cancer. Initially, we established a cisplatin-resistant T24 (CR-T24) bladder cancer cell line. After 2 months of exposure to increasing concentrations of cisplatin, we obtained T24 cells that survived in 2 μM cisplatin (Figure 5A). To compare the AR protein levels between original and CR-T24 cells, we compared their nuclear extracts, since the active form of AR protein occurs only in the nuclear fraction. We used the Lamin B1 antibody as the loading control of the nuclear extract, and for establishing the purity of the fraction, while GAPDH acted as the loading control of the cytosolic fraction. In addition to an increase in the total AR protein level, our study found that the level of active AR protein was higher in the nuclear fraction of CR-T24 when compared to the parental cells (Figure 5B). The protein level of KDM7A in CR-T24 cells decreased in the cytosolic fraction, but remained the same in nuclear fraction. The AR protein level in CR-T24 cells was found to be elevated before and after DHT treatment (Figure 5C), as reported previously [19]. The mRNA levels of several AR target genes also showed an increase, when compared to the parental T24 cells before and after DHT treatment (Figure 5D), suggesting that the elevated AR has functional activity. After confirming an increase in AR activity in CR-T24 cells, we utilized the KDM7A inhibitor TC-E 5002, in conjunction with AR antagonist enzalutamide, to evaluate the role of KDM7A on bladder cancer growth and drug resistance in terms of the AR pathway. Cell viability was tested in parental and CR-T24 cells in the presence of enzalutamide and/or TC-E 5002 (Figure 5E). In order to obtain a clearer picture of the changes in cell viability, we used a non-toxic dose and time of enzalutamide or TC-E 5002 when treated to parental T24 cells. CR-T24 cells were found to be more sensitive to a single treatment with enzalutamide or TC-E 5002 than parental cells. Moreover, when CR-T24 cells were treated with both enzalutamide and TC-E 5002, fewer cells survived compared to the single drug treatments. The anti-cancer effect of this co-treatment was significantly greater in CR-T24 compared to the parental T24 cells. Finally, we investigated the involvement of cellular signaling pathways involved in the anti-cancer effect of AR and/or KDM7A inhibitors on CR-T24 cells. Of the several pathways tested, Akt signaling pathway molecules were significantly decreased upon treatment with AR and/or KDM7A inhibitor. In our experiments, the total and phosphorylated protein levels of Akt and mTOR in T24 cells were decreased after enzalutamide and/or TC-E 5002 treatment. The changes were more significant in CR-T24 compared to the parental cells, and the effect was synergistically increased in co-treatment (Figure 5F; Supplemental Figure S9A). Upon cisplatin treatment, we observed the synergistic effect of two drugs on apoptosis signaling induction in the parental T24 cells, although the resistant cells did not show the apoptosis induction (Supplemental Figure S9B). For studying the changes in cell migration ability in CR-T24 cells, we performed a wound healing assay using both the cell lines treated with AR and/or KDM7A inhibitors (Supplemental Figure S10). The findings showed a decrease in the migration of cells upon treatment with AR and/or KDM7A inhibitors, in both original as well as CR-T24 cells.

### 2.6. KDM7A Knock-Down Attenuated Tumor Growth in Orthotopic Bladder Cancer Xenograft Model

**Figure 6:**
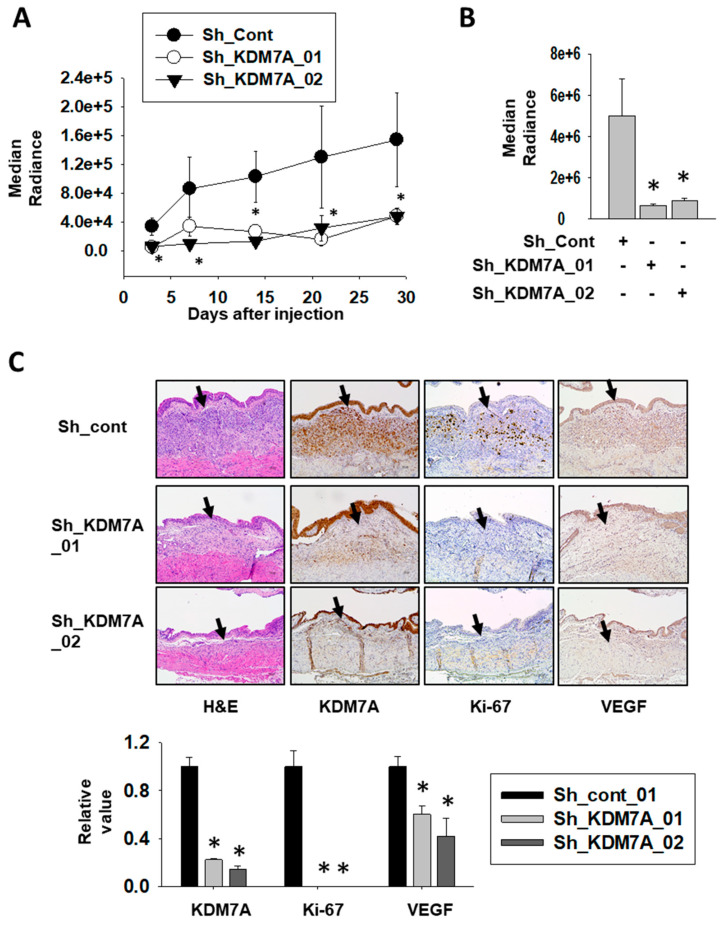
Attenuation of KDM7A expression reduces bladder tumor growth in orthotopic xenograft model. (**A**) Bioluminescent flux plot quantifying tumors in response to control or KDM7A shRNA-expressing T24 cells xenografted into mouse bladder. Error bars represent means ± SEM of each group (*n* = 5). * *p* < 0.05 (Student’s *t*-test) versus the control shRNA (sh-cont) group. (**B**) Bioluminescent flux plot from the extracted bladder from each group. Error bars represent means ± SEM of each group (*n* = 5). * *p* < 0.05 (Student’s *t*-test) versus the sh-cont group. (**C**) Representative hematoxylin-eosin staining and immunohistochemistry images of the orthotopically implanted bladder tumors. Arrow indicates tumor area of the mouse bladder. Positive signal was calculated from at least 3 independent areas, and relative values were plotted. Error bars represent means ± SEM of each group (*n* = 5). * *p* < 0.05 (Student’s *t*-test) versus the sh-cont group.

To investigate the role of KDM7A in bladder tumor growth in vivo, we stably incorporated a luciferase-expressing vector into KDM7A shRNA-expressing bladder cancer cell lines and control cell line. After their inoculation into the bladders of NOD scid gamma (NSG) immune-deficient mice, the growth of the cancer cells was monitored using luciferase signal. The growth of bladder tumors was consistently higher in control cells compared to KMD7A knock-down cells (Figure 6A, Supplemental Figure S11). On the day of sacrifice, the tumors were extracted and the luminescence in control tumors was seen to be significantly higher than in KDM7A knock-down tumors (Figure 6B). Immunostaining of proliferation marker Ki-67 was used to evaluate the aggressiveness of the tumor (Figure 6C). The control tumor was positive for Ki-67, while the KDM7A knock-down tumor was found to be negative. The expression of vascular endothelial growth factor (VEGF) was detected in control tumor, but not in KDM7A knock-down tumors. These results implied that the growth and migration capability of bladder tumor is highly affected by the expression of KDM7A in vivo.

### 2.7. TC-E 5002 Treatment of Xenografted Bladder Tumors Reduced the Tumor Size

**Figure 7:**
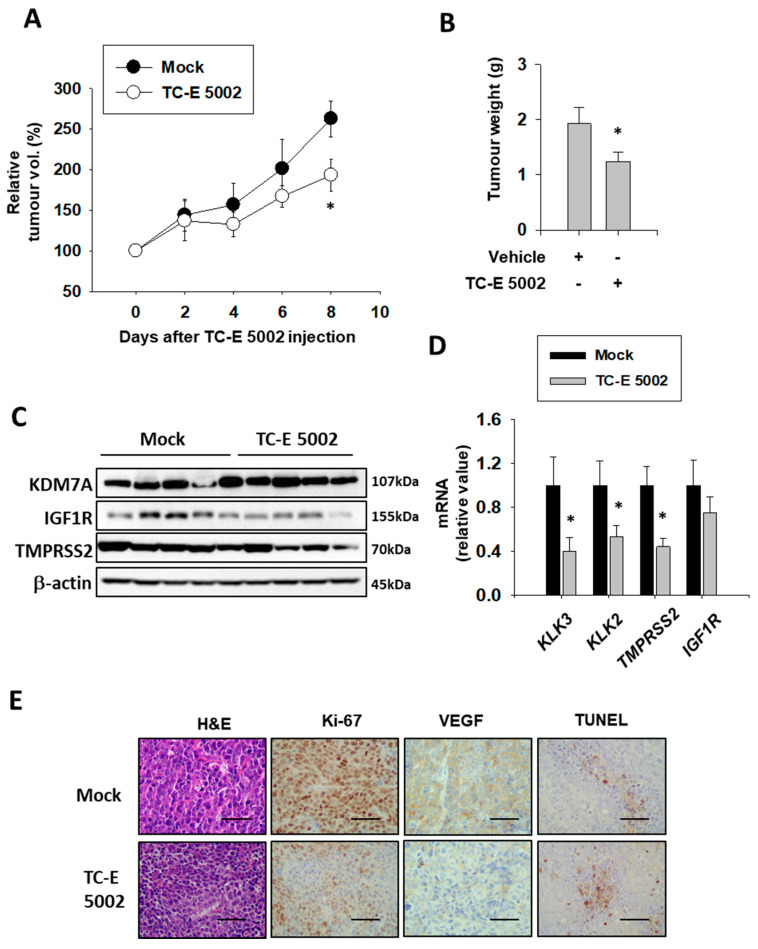
Effect of TC-E 5002 treatment on bladder tumor growth in the xenograft model in NSG mice. (**A**) Relative tumor volume in animals treated with vehicle or TC-E 5002 (10 mg/kg per day). Intraperitoneal drug treatment was started when the average tumor volume reached 200 mm^3^ and continued every day for 8 days. Error bars represent means ± SEM for each group (*n* = 5). * *p* < 0.05 (Student’s *t*-test) versus the vehicle-treated group. (**B**) Weight of tumors excised from animals treated with the vehicle or TC-E 5002. Error bars represent means ± SEM for each group (*n* = 5). * *p* < 0.05 (Student’s *t*-test) versus the vehicle-treated group. (**C**) Total protein was extracted from each xenografted tumor, and Western blotting was performed using the indicated antibodies. (**D**) Total RNA was extracted from each xenografted tumor, and the indicated mRNA levels were measured. Bars represent means ± SEM for each group (*n* = 5). * *p* < 0.05 (Student’s *t*-test) versus the vehicle-treated group. (**E**) Representative hematoxylin-eosin staining and immunohistochemistry images of the excised tumors. Scale bar is 20 μm.

In the prostate study, we found that the KDM7A inhibitor TC-E 5002 effectively reduced tumor cell growth and migration in vitro [32]. In order to assess the in vivo effect of TC-E 5002 on bladder cancer development, we subcutaneously injected the T24 cells in the flanks of the NSG mice. After the tumor volume had reached 200 mm^3^, the mice were divided into 2 groups and injected with the vehicle or TC-E 5002 intraperitoneally daily for 8 days. After day 8, we observed a difference in the tumor sizes between the two groups (Figure 7A). The tumors excised from mice treated with TC-E 5002 weighed lesser than those excised from control mice (Figure 7B). Suppression of the AR activity by TC-E 5002 treatment was evident from the protein and mRNA expression levels of AR-dependent genes in the individual tumors (Figure 7 C,D). In tumors treated with TC-E 5002, the expression of the cell-cycle marker Ki-67 and VEGF protein were decreased, while the expression of the apoptotic DNA-fragmentation marker TUNEL was increased (Figure 7E). Our data exemplified the potential applications of TC-E 5002 in bladder cancer treatment in vivo.

### 2.8. KDM7A Protein and mRNA Level Were Elevated in Bladder Cancer Patients

**Figure 8:**
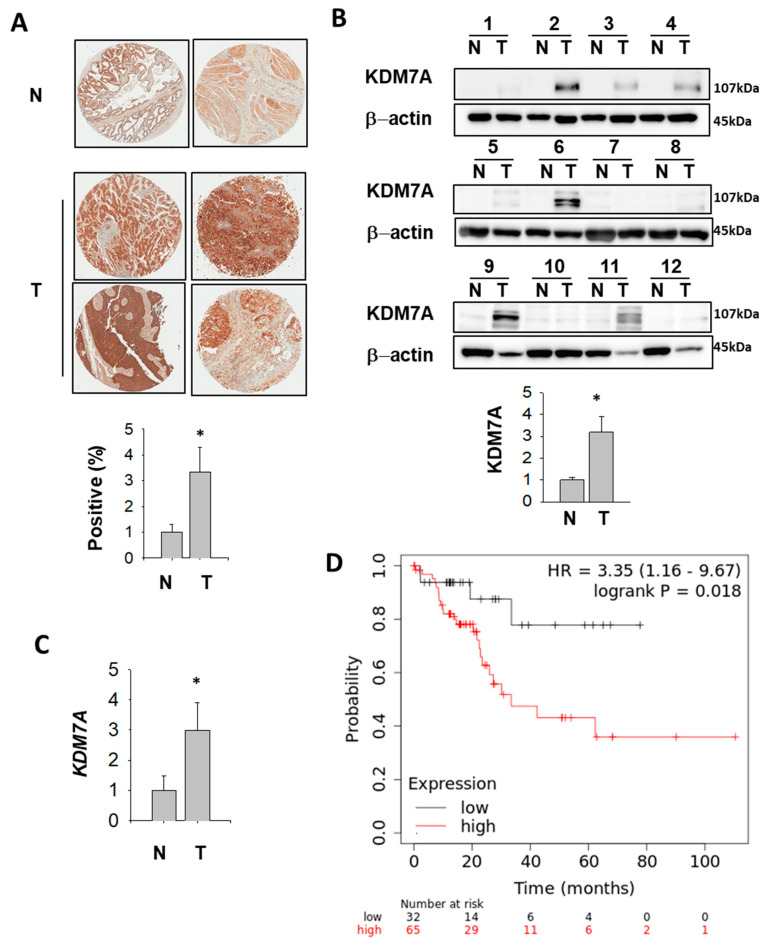
KDM7A is up-regulated in bladder cancer patients (**A**) Representative images of KDM7A expression in bladder tumor and normal tissue arrays (upper figures). N, normal bladder tissue; T, bladder tumor tissue. The expression level of KDM7A from 25 different bladder tumors and 6 normal tissues were calculated and plotted (below graph). * *p* < 0.05 (Student’s *t*-test) between two groups. (**B**) Bladder tumor (T) and adjacent normal (N) tissues were subjected to Western blotting using the KDM7A and beta actin antibodies. Protein bands were analyzed densitometrically and protein levels normalized to beta actin levels were plotted in the lower graph. * *p* < 0.05 (Student’s *t*-test) between two groups. (**C**) Comparison of KDM7A mRNA expression levels between normal and tumor bladder tissues. * *p* < 0.05 (Student’s t-test) between two groups. (**D**) A survival curve was plotted for male bladder cancer patients with cancer stage 2 (*n* = 97). Data were analyzed using the Kaplan–Meier Plotter (www.kmplot.com). Patients with expression above the median are indicated in red line, and patients with expressions below the median in black line. HR means hazard ratio.

In order to analyze KDM7A expression in bladder cancer patients, we performed immunohistochemistry (IHC) of KDM7A using tissue microarray. The results showed a higher level of KMD7A in tumor tissues compared to normal bladder tissue (Figure 8A). In the next step, patient tissue samples were collected, and the KDM7A protein and mRNA levels were analyzed. The protein (Figure 8B, Table 1) and mRNA (Figure 8C, Table 1) levels were significantly higher in tumor samples compared to normal bladder tissue from the same patients. Upon quantification of the signal from Western blot for KDM7A and its comparison with the tumor stages, the results were not statistically significant, which might be due to the small sample size (Supplemental Figure S12). Next, we evaluated the correlation between mRNA expression level of KDM7A and clinical outcome using a Kaplan–Meier plotter in public database (www.kmplot.com). We examined the prognostic value of KDM7A expression at each tumor stage, and in both men and women, in the bladder cancer database using KDM7A as the ‘key gene’ for data mining. High *KDM7A* mRNA expression was associated with significantly worse overall survival (OS) in men with stage 2 bladder cancer (Figure 8D). However, we were not able to identify a correlation in other stages of male cancer patients (Supplemental Figures S13 and S14), or in any stages of female patients.

## 3. Discussion

Although the epigenetic regulation of AR has been extensively studied in prostate cancer, a growing body of evidence has suggested a role for AR in other cancers, including colon, breast, and bladder cancer. Because anti-cancer drugs targeting AR have been well-characterized in prostate cancer, existing drugs can be explored as potential treatment options for other AR-positive cancers as well. In this paper, we focused on the AR function in bladder cancer, because of its high malignant character, which is known to be related to AR malfunction.

KDM7A histone demethylase is known to act on H3K27 residues, which function as repressive marks of transcription. Consequently, lowering KDM7A activity results in H3K27 methylation on chromatin and reduced gene transcription. We had previously showed that AR binding to KLK3, KLK2, and TMPRSS2 gene promoters was decreased in KDM7A knock-down prostate cells, and the expression levels of these genes were also reduced. More importantly, the effect of the KDM7A inhibitor TC-E 5002 on prostate cancer cell proliferation was analyzed, and we observed that prostate cancer cell growth was reduced on treatment with TC-E 5002 treatment. In the present study, we found that the bladder cancer cells expressing KDM7A shRNA also showed decreased cell proliferation. As expected, the AR expression in bladder cancer cells was lower than that of prostate cells (Supplemental Figure S1). Therefore, the effect of KDM7A knockdown was not as dramatic as in prostate cells. However, the reduction of bladder cancer cell growth, migration and metastatic abilities was found to be statistically significant. On the other hand, when we used TC-E 5002, a chemical inhibitor of KDM7A, we detected a relatively small effect on T24 bladder cancer cell growth (Figure 5E) compared to the inhibition effect of KDM7A knock-down (Figure 3A). This could be because the selected TC-E 5002 treatment time and concentration were not high enough to kill the original T24 cells, since we wanted to see the effect of TC-E 5002 and/or enzalutamide on CR-T24 cells. When we continued TC-E 5002 treatment for longer durations and using higher doses, more cell death was achieved. Since many in vitro studies have shown the anti-cancer effect of enzalutamide in bladder cancer, including drug-resistant conditions [16,17,18,19], co-treatment of TC-E 5002 together with enzalutamide was performed to study the effect on cisplatin-resistant bladder cancer cells. As we can see in the right part of graph in Figure 5E, CR-T24 cells, which had elevated AR expression, died more efficiently upon TC-E 5002 treatment. In addition to cisplatin-resistant T24 [19], gemcitabine-resistant T24 has also been reported to show increased AR expression [17]. Therefore, it would be interesting to investigate whether treatment of gemcitabine-resistant cells with TC-E 5002 has the same effect. In particular, the effect of co-treatment with enzalutamide and TC-E 5002 on CR-T24 bladder cancer cell line could be useful for understanding the value of this treatment in drug-resistant bladder cancer. In addition to testing TC-E 5002 in vitro in cell culture, our in vivo data using the xenograft bladder tumors illustrated the future possibility of applying the drug clinically.

Although we focused only on demonstrating the role of KDM7A in regulating AR activity, we cannot rule out the possibility that KDM7A acts on other factors, regulating the cell cycle, or that our results are owing to the non-specific knockdown effect of KDM7A. This is because the AR expression level of the cell lines that we used was notably lower than that of prostate cancer, and the repressive effect on neoplasia was relatively strong. Factors other than AR may include KLF4 and c-MYC, which were found to be involved in breast cancer stem cell maintenance [34]. The use of only two bladder cell lines with undifferentiated character may also limit the universality of our data. Therefore, it would be interesting to study the function of KDM7A in other differentiated cancer cells.

Our finding that the protein and mRNA levels of KDM7A are increased in bladder cancer tissues (Figure 8) may point to elevated AR activity in the cancer. Based on our results, it is possible that KDM7A controls AR activity as an epigenetic co-activator during cancer progression. Most importantly, the KDM7A protein level increased in tumor tissues compared to matching normal tissues (Figure 8B). However, the correlation of KDM7A expression level with each cancer stage was not statistically significant, perhaps due to the small number of cases for each stage (Supplemental Figure S12). Nonetheless, the fact that we identified a correlation between high KDM7A mRNA levels and cancer-dependent deaths only in men (Figure 8D), but not in women, may explain the AR dependency of this effect. Interestingly, this correlation was lost in men with higher grades of cancer (grade 3 and 4; Supplement Supplement Figures S13 and S14), which may be due to the AR loss-of-function during the progression of bladder cancer. Given that this phenomenon is widely reported in prostate cancer, a similar mechanism may exist in bladder cancer. For the early stage male bladder cancer patients, it would be interesting to explore whether an AR antagonist can be used as an anti-cancer drug upon screening KMD7A expression levels. In addition, the regulation of other transcription factors besides AR, which are under the control of KDM7A, should be considered.

An increasing number of histone-modifying enzymes have been shown to be important for bladder cancer development. Among them, the knock-down of LSD1 was found to effectively repress bladder cancer growth, and this effect was confirmed to be associated with AR activity regulation [47]. Additionally, it has been shown that the up-regulation of histone methyl transferase SMYD3 promotes bladder cancer progression. It is worth noting that, although the authors demonstrated that SMYD3 physically interacted with the BCLAF1 promoter [48], it is possible that SMYD3 may regulate bladder cancer growth *via* AR, because of its previously reported interaction with the receptor [30]. Our data point to the possibility that KDM7A may regulate AR in bladder cancer together with the above-mentioned co-regulators. Investigating potential interactions of the above-mentioned co-factors with KDM7A on the AR-regulated gene promoters would lead to a better understanding of the mechanism.

The anti-cancer effect of many histone methylase or demethylase inhibitors have been reported in bladder cancer, and many of them are presently being developed for cancer treatment [49]. Based on our data, we suggest that KDM7A inhibitor TC-E 5002 could be added to this list. Although further in-depth research is needed to validate the results of our study, our findings suggest that KDM7A could be a new target for treating bladder cancer and overcoming drug resistance, in conjunction with an AR inhibitor.

## 4. Materials and Methods

### 4.1. Materials

RPMI-1640, DMEM, trypsin, anti-biotics, Trizol and Lipofectamine 2000 were purchased from Invitrogen (Carlsbad, CA, USA). Fetal bovine serum and culture media were obtained from HyClone Laboratories Inc. (South Logan, UT, USA). The detailed information of all primary antibodies is listed in Supplemental Table S1.

### 4.2. Cell Lines, Plasmids, Virus Production and Infection

The T24, J82, and 293T cell lines were purchased from the American Type Culture Collection (Rockville, MD). T24 and J82 cells were cultured in RPMI-1640, and 293T cells for lentiviral package were cultured in DMEM medium at 37 °C in 5% CO_2_, which was supplemented with 10% fetal bovine serum. For gene silencing, the control or KDM7A shRNA expressing lenti-virus packaging and stable cell line establishment were performed as described [32]. The oligo sequence used for KDM7A shRNA 01 cloning is 5′-CCGGTGGATTTGATGTCCCTATTATCTCGAGATAATAGGGACATCAAATCCAT TTTT-3′, and for shRNA 02 sequence is 5′-CCGGTTAGACCTGGACACCTTATTACTCGAGTAATAA GGTGTCCAGGTCTAATTTTT-3′. The oligo sequence for control shRNA cloning is 5′-CCGGCGTGA TCTTCACCGACAAGATCTCGAGATCTTGTCGGTGAAGATCACGTTTTT-3′. FUGW-luc vector (from the Molecular Imaging and Neurovascular Research Laboratory, Dongguk University Ilsan Hospital, Goyang, Korea) expressing cells were produced as described below. FUGW-luc vector was cut with XhoI enzyme and transfected into the T24 cell line, expressing either control vector or KMD7A shRNA vector. The cells with FUGW-luc incorporation were sorted with GFP channel using BD FACSAria II (BD Biosciences, Franklin Lakes, NJ, USA).

### 4.3. Colony Formation Assay and Cell Viability Assay

For the colony formation assay, 1000 cells were plated in 6-well plates. The cells were cultured for 14 days and stained with 0.1% crystal violet. The cell colonies were photographed, and the number of colonies comprising more than 50 individual cells was counted using SZX7 stereo microscope (Olympus, Tokyo, Japan). For the cell viability assay, cells (2000 to 3000 cells/well) were dispensed in 100 μL culture medium in a 96-well plate, and incubated for the indicated time. EZ-Cytox cell viability kit (Daeil-Lab, Seoul, Korea) solution (10 μL) was mixed with the culture medium in each well of the plate. Samples were incubated for 1 h at 37 °C, and the absorbance of each sample at 450 nm was measured using a microplate reader (PerkinElmer, Waltham, MA, USA).

### 4.4. RNA Isolation and the Real-Time Quantitative Polymerase Chain Reaction (RT-qPCR)

The total cellular RNA was extracted using the Trizol reagent (Ambion, Austin, TX, USA), according to the manufacturer’s instructions. For the induction of AR activity, 5 nM 5α-dihydrotestosterone (DHT) was added after one day of serum deprivation. For each reverse-transcription reaction, 1 μg of total RNA was used for cDNA synthesis, using the MultiScribe Reverse Transcription Kit from Life Technologies (Carlsbad, CA, USA). RT-qPCR was performed using the EvaGreen qPCR Master Mix Kit from Applied Biological Materials Inc. (Richmond, BC, Canada) and a StepOne^TM^ Real-Time PCR System (Applied Biosystems, Foster City, CA, USA). The quantity of 18S ribosomal RNA was measured as an internal control. The sequences of the primers used for RT-qPCR are listed in Supplemental Table S2.

### 4.5. Wound Healing and Cell Invasion Assays

The wound healing assay was performed on 100% confluent cells, plated into 6-well culture plates. Straight scratches were made by using a pipette tip. The cells were washed twice to remove debris, followed by the addition of fresh medium. The cells were incubated in a 5% CO_2_ environment at 37 °C, and observed using a SZX7 stereo microscope at the indicated time. The scratched areas were measured using ImageJ program (ver. 1.43u; www.rsb.info.nih.gov/ij). For the invasion assay, cells (5 × 10^4^/well) were plated in the upper chambers of Transwells without serum, using Matrigel-coated polycarbonate membranes (Corning, Big Flats, NY, USA). The basal medium containing 10% fetal bovine serum was added into the lower chambers, as a chemoattractant for cell migration. After 48-h, non-migrated cells were removed from the upper chambers, while cells that migrated through chambers were fixed using 10% ethanol (Sigma-Aldrich). After cells were stained with the 0.01% crystal violet solution (Sigma-Aldrich), migrated cells were randomly counted in five different microscopic fields at 20× magnification.

### 4.6. Human Ethics Approval and Collection of Human Tissues

The frozen tissues from bladder cancer patients were collected from Seoul Nation University Hospital Tissue Bank, with the approval of Institutional Review Board No. H-1004-037-315 (Date of approval: 06/11/2010). The demographic data of each patient are shown in Table 1. Tumor tissues and matching normal tissues from the same patient were identified from the pathology results. For Western blotting, 50–200 mg of tissues was ground in liquid nitrogen and lysed with RIPA buffer.

### 4.7. Animal Studies and In Vivo Bioluminescent Imaging

All animal experiments were performed in accordance with the Seoul National University Hospital institutional guidelines, under IACUC protocol No.16-0167-C2A0 (Date of approval: 07/20/2018). NOD scid gamma (NSG) mice were bred and maintained under specific pathogen-free (SPF) conditions. For generating orthotopic tumors, 1 × 10^5^ T24 human bladder cells expressing the indicated shRNA and Luciferase expression vector were injected into the bladder of six-week-old male NSG mice (*n* = 5 for each group). For injection, the cells were suspended with 100 μL of 50 % Matrigel (BD Biosciences) in complete media. The mice from each group were injected intraperitoneally with 150 mg/kg D-luciferin (Promega, Madison, WI, USA), 15 min before acquiring the image. After anesthetizing the mice using 1–3% isoflurane, the photons emitted from the tumor were detected with Xenogen IVIS imaging system 200 (Alameda, CA, USA) as described. The image acquisition period was 1 s. Living Image (Version 2.20, Xenogen) was used to quantify signals emitted from the regions of interest. The mice were sacrificed after 30 days of tumor implantation, and bladder tumor was fixed in 4% paraformaldehyde at 4 °C and embedded in paraffin. The specimens were subjected to IHC with the indicated antibodies. For subcutaneous xenografting, six-week-old male NSG mice were injected, in their lower flanks, with 1 × 10^7^ T24 cells in 100 μL of 50% (*v*/*v*) Matrigel (*n* = 5 for each group). When the average tumor volume reached 200 mm^3^, mice were randomly assigned into two groups and injected intraperitoneally everyday with vehicle (0.05 mL; 90% corn-oil and 10% DMSO (*v*/*v*)) or 10 mg/kg TC-E 5002 in the same vehicle, respectively. The tumors were measured every other day, and at the end of 8 days of treatment, the mice were sacrificed, and the tumors were excised, weighed, and either frozen or fixed in formalin for further analyses.

### 4.8. Western Blotting

The cells (5 × 10^6^) and ground tissue (50–200 mg) were lysed in 1ml RIPA buffer (150 mM NaCl, 50 mM Tris-HCl [pH 7.2], 0.5% NP-40, 1% Triton X-100, and 1% sodium deoxycholate), containing a protease/phosphatase inhibitor cocktail (Sigma-Aldrich, St. Louis, MO, USA). For the induction of AR activity, 5 nM 5α-dihydrotestosterone (DHT) was added after one day of serum deprivation. The cell lysates were separated on sodium dodecyl sulfate-polyacrylamide gels and transferred to an Immobilon-P membrane (Millipore, Darmstadt, Germany). The membranes were blocked with 5% skim milk in 0.1% Tween-20 for 1 h, followed by overnight incubation at 4 °C with the indicated primary antibodies. The membranes were incubated with a horseradish peroxidase-conjugated secondary antibody (1:5000) for 1 h and developed using the ECL-Plus Kit (Thermo Scientific, Rockford, IL, USA).

### 4.9. Immunohistochemical Staining and Analysis

Bladder cancer tissue microarrays purchased from SuperBioChips Laboratories (Seoul, Korea) were stained with an anti-KDM7A antibody. The slides were incubated with an anti-Rabbit IgG secondary antibody and hematoxylin and eosin (nuclear staining dye). The expression level of KDM7A from 25 different bladder tumors and 6 normal tissues was calculated and plotted. The expression of KDM7A positive cells was evaluated using the Cytoplasmic V2.0 algorithm in Aperio ImageScope software (Leica, Nussloch, Germany), and logistic regression analysis was used to compare the expression patterns between groups. Mouse tumor tissues were fixed in paraffin after formaldehyde fixation. Mouse tissue slides were deparaffinized and stained with indicated antibodies, and the slides were photographed under a Leica microscope (Wetzlar, Germany). Positive signals were counted from at least 3 different fields of the same area, and relative values were calculated.

### 4.10. The Kaplan–Meier Plotter

The prognostic significance of the mRNA expression of KDM7A was evaluated using the Kaplan–Meier plotter (www.kmplot.com), an online database comprising gene expression data and clinical data. In order to assess the prognostic value of the KDM7A gene, the patient samples were divided into two cohorts according to the median expression level of the gene (high vs. low expression). We analyzed overall survival (OS) of bladder cancer patients by the Kaplan–Meier survival plot. KDM7A gene was uploaded into the database to obtain the Kaplan–Meier survival plot, in which the number-at-risk was shown below the main plot. Log rank p-value and hazard ratio (HR) with 95% confidence intervals were calculated and displayed on the webpage. We exported the plot data as a PowerPoint file.

### 4.11. Statistical Analyses

All data were analyzed using Microsoft Excel 2010 software, unless otherwise stated. Continuous variables were analyzed using Student’s *t*-test if the data were normally distributed. All statistical tests were two-sided. Differences were considered significant in cases where *p* values were <0.05.

## Figures and Tables

**Table 1:** Demographics of patients used for tissue extract.

No.	Age	Sex	T Stage
1	66	M	T2bN0(0/21) LVI necrosis
2	73	F	T2aN0(0/16)
3	58	M	TaN0(0/25) CIS
4	56	F	T1N0(0/37)
5	84	M	T4aN0(0/1) LVI, Perineural invasion
6	67	M	TaN0(0/26)
7	64	M	T3aN2(2/7) LVI, Perineural invasion
8	75	M	T3bN1(1/17), Perineural invasion
9	72	M	T2aN0(0/14)
10	70	M	T3aN0(0/15) Lymphatic invasion
11	61	M	T4aN2(3/17), LVI Perineural invasion, necrosis
12	63	M	T3aN0(0/13)

## Supplementary Materials

Supplementary materials can be found at https://www.mdpi.com/1422-0067/21/16/5658/s1. Figure S1. AR expression in bladder cell lines. Figure S2. AR expression levels in KDM7A knockdown J82 cells. Figure S3. The primer positions for ChIP-qPCR in the indicated gene promoters are illustrated. Figure S4. AR inhibition reduced cell viability of bladder cancer cells. Figure S5. T24 and J82 bladder cancer cells expressing KDM7A shRNAs or treated with TC-E 5002 photographed under phase contrast microscope. Figure S6. Enzalutamide treatment reduced cell mobility in bladder cancer cells. Figure S7. KDM7A knock-down reduced cell mobility in bladder cancer cells. Figure S8. Protein bands from Figure 4C were analyzed densitometrically and protein levels were normalized to beta-actin levels. Figure S9. The enzalutamide and TC-E 5002 treatment reduces cell growth and increased apoptosis in cisplatin resistant T24 cells. Figure S10. Enzalutamide and TC-E 5002 treatment reduced cell mobility in bladder cancer cells. Figure S11. IVIS images demonstrating tumor formation on the day of sacrifice. Figure S12. KDM7A protein expression in human bladder tumor tissues and normal tissues from the same patient. Figure S13. Survival curve was plotted for male bladder cancer patients with cancer stage 3. Figure S14. A survival curve was plotted for male bladder cancer patients with cancer stage 4. Table S1. The company and catalog numbers of antibodies. Table S2. Oligonucleotide sequences for RT-PCR and ChIP-PCR.

## Abbreviations

AR	androgen receptor
BCa	bladder cancer
CBP	CREB-binding protein
ChIP	chromatin immunoprecipitation
DHT	dihydrotestosterone
ECAD	E-cadherin
EMT	epithelial-mesenchymal transition
EZH2	enhancer of zeste homolog 2
HAT	histone acetyltransferase
IGF1R	insulin-like growth factor 1 receptor
KDM	lysine demethylase
KLK	kallikrein related peptidase
LSD1	lysine-specific histone demethylase 1
NCAD	N-cadherin
PCAF	P300/CBP-associated factor
PSA	prostate specific antigen
PHD	plant homeodomain
SET	su(var)3-9, enhancer-of-zeste and trithorax
SRC	steroid receptor coactivator proteins
VEGF	vascular endothelial growth factor

## References

[1] Siegel R.L., Miller K.D., Jemal A. (2019). Cancer statistics, 2019. CA Cancer J. Clin..

[2] Sievert K.D., Amend B., Nagele U., Schilling D., Bedke J., Horstmann M., Hennenlotter J., Kruck S., Stenzl A. (2009). Economic aspects of bladder cancer: What are the benefits and costs?. World J. Urol..

[3] Dobruch J., Daneshmand S., Fisch M., Lotan Y., Noon A.P., Resnick M.J., Shariat S.F., Zlotta A.R., Boorjian S.A. (2016). Gender and Bladder Cancer: A Collaborative Review of Etiology, Biology, and Outcomes. Eur. Urol..

[4] Chen J., Cui Y., Li P., Liu L., Li C., Zu X. (2017). Expression and clinical significance of androgen receptor in bladder cancer: A meta-analysis. Mol. Clin. Oncol..

[5] Claps M., Petrelli F., Caffo O., Amoroso V., Roca E., Mosca A., Maines F., Barni S., Berruti A. (2018). Testosterone Levels and Prostate Cancer Prognosis: Systematic Review and Meta-analysis. Clin. Genitourin. Cancer.

[6] Sumanasuriya S., De Bono J. (2018). Treatment of Advanced Prostate Cancer-A Review of Current Therapies and Future Promise. Cold Spring Harb. Perspect. Med..

[7] Roshan M.H., Tambo A., Pace N.P. (2016). The role of testosterone in colorectal carcinoma: Pathomechanisms and open questions. EPMA J..

[8] Ricciardelli C., Bianco-Miotto T., Jindal S., Butler L.M. (2018). The Magnitude of Androgen Receptor Positivity in Breast Cancer is Critical for Reliable Prediction of Disease Outcome. Clin. Cancer Res..

[9] Zhang B.G., Du T., Zang M.D., Chang Q., Fan Z.Y., Li J.F., Yu B.Q., Su L.P., Li C., Yan C. (2014). Androgen receptor promotes gastric cancer cell migration and invasion via AKT-phosphorylation dependent upregulation of matrix metalloproteinase 9. Oncotarget.

[10] Li Y., Izumi K., Miyamoto H. (2012). The role of the androgen receptor in the development and progression of bladder cancer. Jpn. J. Clin. Oncol..

[11] Zhuang Y.H., Blauer M., Tammela T., Tuohimaa P. (1997). Immunodetection of androgen receptor in human urinary bladder cancer. Histopathology.

[12] Boorjian S., Ugras S., Mongan N.P., Gudas L.J., You X., Tickoo S.K., Scherr D.S. (2004). Androgen receptor expression is inversely correlated with pathologic tumor stage in bladder cancer. Urology.

[13] Boorjian S.A., Heemers H.V., Frank I., Farmer S.A., Schmidt L.J., Sebo T.J., Tindall D.J. (2009). Expression and significance of androgen receptor coactivators in urothelial carcinoma of the bladder. Endocr.-Relat. Cancer.

[14] Miyamoto H., Yang Z., Chen Y.-T., Ishiguro H., Uemura H., Kubota Y., Nagashima Y., Chang Y.-J., Hu Y.-C., Tsai M.-Y. (2007). Promotion of Bladder Cancer Development and Progression by Androgen Receptor Signals. JNCI J. Natl. Cancer Inst..

[15] Hsu J.-W., Hsu I., Xu D., Miyamoto H., Liang L., Wu X.-R., Shyr C.-R., Chang C. (2013). Decreased Tumorigenesis and Mortality from Bladder Cancer in Mice Lacking Urothelial Androgen Receptor. Am. J. Pathol..

[16] Kawahara T., Ide H., Kashiwagi E., El-Shishtawy K.A., Li Y., Reis L.O., Zheng Y., Miyamoto H. (2016). Enzalutamide inhibits androgen receptor-positive bladder cancer cell growth. Urol. Oncol..

[17] Kameyama K., Horie K., Mizutani K., Kato T., Fujita Y., Kawakami K., Kojima T., Miyazaki T., Deguchi T., Ito M. (2017). Enzalutamide inhibits proliferation of gemcitabine-resistant bladder cancer cells with increased androgen receptor expression. Int. J. Oncol..

[18] Kawahara T., Inoue S., Kashiwagi E., Chen J., Ide H., Mizushima T., Li Y., Zheng Y., Miyamoto H. (2017). Enzalutamide as an androgen receptor inhibitor prevents urothelial tumorigenesis. Am. J. Cancer Res..

[19] Kashiwagi E., Ide H., Inoue S., Kawahara T., Zheng Y., Reis L.O., Baras A.S., Miyamoto H. (2016). Androgen receptor activity modulates responses to cisplatin treatment in bladder cancer. Oncotarget.

[20] Baylin S.B. (2005). DNA methylation and gene silencing in cancer. Nat. Clin. Pract. Oncol..

[21] Dompe C., Janowicz K., Hutchings G., Moncrieff L., Jankowski M., Nawrocki M.J., Józkowiak M., Mozdziak P., Petitte J., Shibli J.A. (2020). Epigenetic Research in Stem Cell Bioengineering-Anti-Cancer Therapy, Regenerative and Reconstructive Medicine in Human Clinical Trials. Cancers.

[22] Black J.C., Van Rechem C., Whetstine J.R. (2012). Histone lysine methylation dynamics: Establishment, regulation, and biological impact. Mol. Cell.

[23] Greer E.L., Shi Y. (2012). Histone methylation: A dynamic mark in health, disease and inheritance. Nat. Rev. Genet..

[24] Kooistra S.M., Helin K. (2012). Molecular mechanisms and potential functions of histone demethylases. Nat. Rev. Mol. Cell Biol..

[25] Baumgart S.J., Haendler B. (2017). Exploiting Epigenetic Alterations in Prostate Cancer. Int. J. Mol. Sci..

[26] Kahl P., Gullotti L., Heukamp L.C., Wolf S., Friedrichs N., Vorreuther R., Solleder G., Bastian P.J., Ellinger J., Metzger E. (2006). Androgen receptor coactivators lysine-specific histone demethylase 1 and four and a half LIM domain protein 2 predict risk of prostate cancer recurrence. Cancer Res..

[27] Coffey K., Rogerson L., Ryan-Munden C., Alkharaif D., Stockley J., Heer R., Sahadevan K., O’Neill D., Jones D., Darby S. (2013). The lysine demethylase, KDM4B, is a key molecule in androgen receptor signalling and turnover. Nucleic Acids Res..

[28] Han M., Xu W., Cheng P., Jin H., Wang X. (2017). Histone demethylase lysine demethylase 5B in development and cancer. Oncotarget.

[29] Deb G., Thakur V.S., Gupta S. (2013). Multifaceted role of EZH2 in breast and prostate tumorigenesis. Epigenetics.

[30] Liu C., Wang C., Wang K., Liu L., Shen Q., Yan K., Sun X., Chen J., Liu J., Ren H. (2013). SMYD3 as an oncogenic driver in prostate cancer by stimulation of androgen receptor transcription. J. Natl. Cancer Inst..

[31] Mounir Z., Korn J.M., Westerling T., Lin F., Kirby C.A., Schirle M., McAllister G., Hoffman G., Ramadan N., Hartung A. (2016). ERG signaling in prostate cancer is driven through PRMT5-dependent methylation of the Androgen Receptor. Elife.

[32] Lee K.H., Hong S., Kang M., Jeong C.W., Ku J.H., Kim H.H., Kwak C. (2018). Histone demethylase KDM7A controls androgen receptor activity and tumor growth in prostate cancer. Int. J. Cancer.

[33] Tsukada Y., Fang J., Erdjument-Bromage H., Warren M.E., Borchers C.H., Tempst P., Zhang Y. (2006). Histone demethylation by a family of JmjC domain-containing proteins. Nature.

[34] Meng Z., Liu Y., Wang J., Fan H., Fang H., Li S., Yuan L., Liu C., Peng Y., Zhao W. (2020). Histone demethylase KDM7A is required for stem cell maintenance and apoptosis inhibition in breast cancer. J. Cell. Physiol..

[35] Yang X., Wang G., Wang Y., Zhou J., Yuan H., Li X., Liu Y., Wang B. (2019). Histone demethylase KDM7A reciprocally regulates adipogenic and osteogenic differentiation via regulation of C/EBPalpha and canonical Wnt signalling. J. Cell. Mol. Med..

[36] Higashijima Y., Matsui Y., Shimamura T., Nakaki R., Nagai N., Tsutsumi S., Abe Y., Link V.M., Osaka M., Yoshida M. (2020). Coordinated demethylation of H3K9 and H3K27 is required for rapid inflammatory responses of endothelial cells. EMBO J..

[37] Grad J.M., Le Dai J., Wu S., Burnstein K.L. (1999). Multiple Androgen Response Elements and a Myc Consensus Site in the Androgen Receptor (AR) Coding Region Are Involved in Androgen-Mediated Up-Regulation of AR Messenger RNA. Mol. Endocrinol..

[38] Blackburn J., Vecchiarelli S., Heyer E.E., Patrick S.M., Lyons R.J., Jaratlerdsiri W., van Zyl S., Bornman M.S.R., Mercer T.R., Hayes V.M. (2019). TMPRSS2-ERG fusions linked to prostate cancer racial health disparities: A focus on Africa. Prostate.

[39] Kim J., Coetzee G.A. (2004). Prostate specific antigen gene regulation by androgen receptor. J. Cell. Biochem..

[40] Lai J., Myers S.A., Lawrence M.G., Odorico D.M., Clements J.A. (2009). Direct Progesterone Receptor and Indirect Androgen Receptor Interactions with the Kallikrein-Related Peptidase 4 Gene Promoter in Breast and Prostate Cancer. Mol. Cancer Res..

[41] Schayek H., Seti H., Greenberg N.M., Sun S., Werner H., Plymate S.R. (2010). Differential regulation of insulin-like growth factor-I receptor gene expression by wild type and mutant androgen receptor in prostate cancer cells. Mol. Cell. Endocrinol..

[42] Eisermann K., Broderick C.J., Bazarov A., Moazam M.M., Fraizer G.C. (2013). Androgen up-regulates vascular endothelial growth factor expression in prostate cancer cells via an Sp1 binding site. Mol. Cancer.

[43] Gao L., Schwartzman J., Gibbs A., Lisac R., Kleinschmidt R., Wilmot B., Bottomly D., Coleman I., Nelson P., McWeeney S. (2013). Androgen receptor promotes ligand-independent prostate cancer progression through c-Myc upregulation. PLoS ONE.

[44] Xu Y., Chen S.Y., Ross K.N., Balk S.P. (2006). Androgens induce prostate cancer cell proliferation through mammalian target of rapamycin activation and post-transcriptional increases in cyclin D proteins. Cancer Res..

[45] Li Y., Zhang D.Y., Ren Q., Ye F., Zhao X., Daniels G., Wu X., Dynlacht B., Lee P. (2012). Regulation of a novel androgen receptor target gene, the cyclin B1 gene, through androgen-dependent E2F family member switching. Mol. Cell. Biol..

[46] Frezza M., Yang H., Dou Q.P. (2011). Modulation of the tumor cell death pathway by androgen receptor in response to cytotoxic stimuli. J. Cell. Physiol..

[47] Kauffman E.C., Robinson B.D., Downes M.J., Powell L.G., Lee M.M., Scherr D.S., Gudas L.J., Mongan N.P. (2011). Role of androgen receptor and associated lysine-demethylase coregulators, LSD1 and JMJD2A, in localized and advanced human bladder cancer. Mol. Carcinog..

[48] Shen B., Tan M., Mu X., Qin Y., Zhang F., Liu Y., Fan Y. (2016). Upregulated SMYD3 promotes bladder cancer progression by targeting BCLAF1 and activating autophagy. Tumour Biol. J. Int. Soc. Oncodev. Biol. Med..

[49] Song Y., Wu F., Wu J. (2016). Targeting histone methylation for cancer therapy: Enzymes, inhibitors, biological activity and perspectives. J. Hematol. Oncol..

