# Supplementary material for: Histone Demethylase KDM7A Regulates Androgen Receptor Activity, and Its Chemical Inhibitor TC-E 5002 Overcomes Cisplatin-Resistance in Bladder Cancer Cells
Source: Int J Mol Sci. 2020 Aug 6;21(16):5658. doi: 10.3390/ijms21165658 (PMC7460860; doi:10.3390/ijms21165658)
Supplement: Supplementary file 1 [file ijms-21-05658-s001.pdf]

Supplementary Material

A

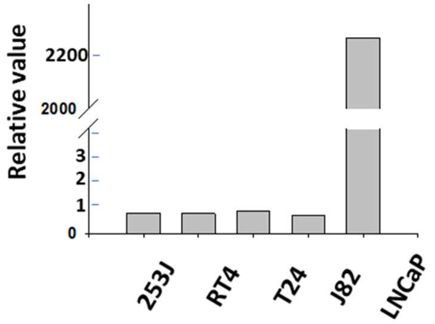

cell line	AR mRNA
253J	1
RT4	0.965756
T24	1.100978
J82	0.857794
LNCaP	2268.702

B

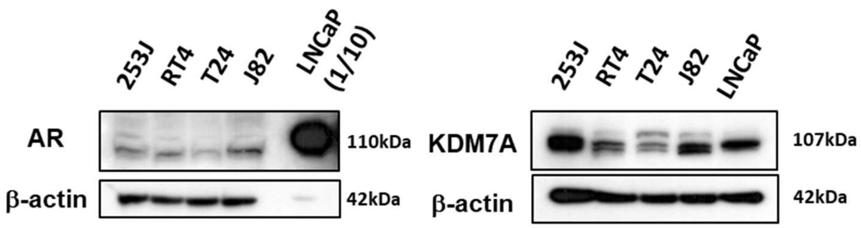

C

	KLK3	KLK4	TMPRSS2	IGF1R	VEGF	MYC
253J	1	1	1	1	1	1
RT4	3.264058	2.945134	2005.853	3.418429	2.485151	0.523647
T24	0.730353	3.806152	4.179509	0.487452	0.484085	0.593231
J82	0.592546	2.11648	0.280616	0.447513	0.481854	0.511687
LNCaP	7332.045	70.93046	8344.828	1.286395	0.720298	2.013911

Figure S1. AR expression in bladder cell lines. (**A**) mRNA levels of AR in various bladder cancer cells and prostate cancer cell LNCaP. The relative mRNA levels from each cells are listed in the right table. (**B**) protein level of AR and KDM7A in various bladder cancer cells and prostate cancer cell LNCaP. Same amount of bladder cancer cell extracts were compared with 1/10 amount of LNCaP cell extract for AR western blotting. Same amount of all listed cell lines were compared for KDM7A western blotting. (**C**) Relative mRNA levels of AR target genes in various bladder cancer cells and prostate cancer LNCaP.

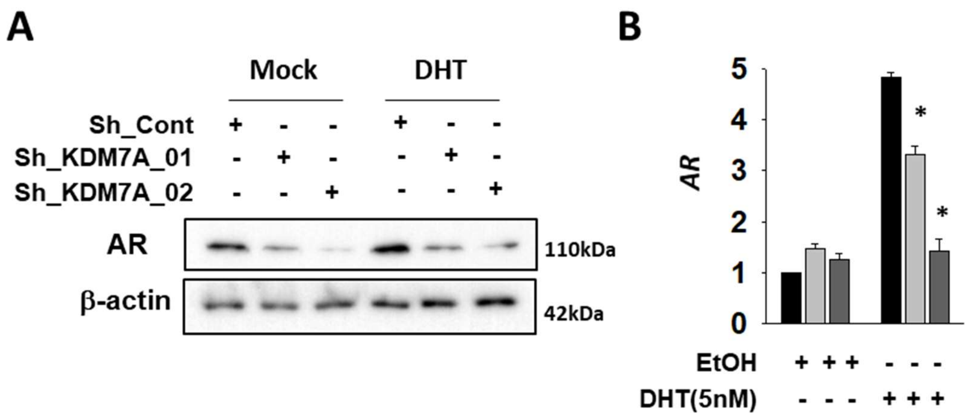

Figure S2. AR expression levels in KDM7A knockdown J82 cells. (**A**) The AR protein levels in KDM7A knock-down J82 cells treated with DHT were analyzed with indicated antibodies. (**B**) The AR mRNA levels in J82 cells after DHT induction were measured by RT-qPCR . Bars represent the means ± SD of three independent experiments, and * denotes $p < 0.05$ (student t-test) versus the control shRNA (sh-cont) group.

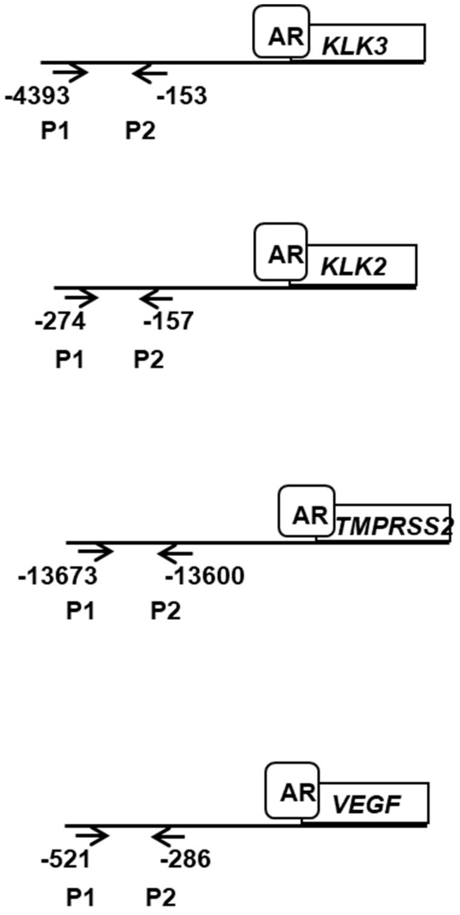

Figure S3. The primer positions for ChIP-qPCR in the indicated gene promoters are illustrated.

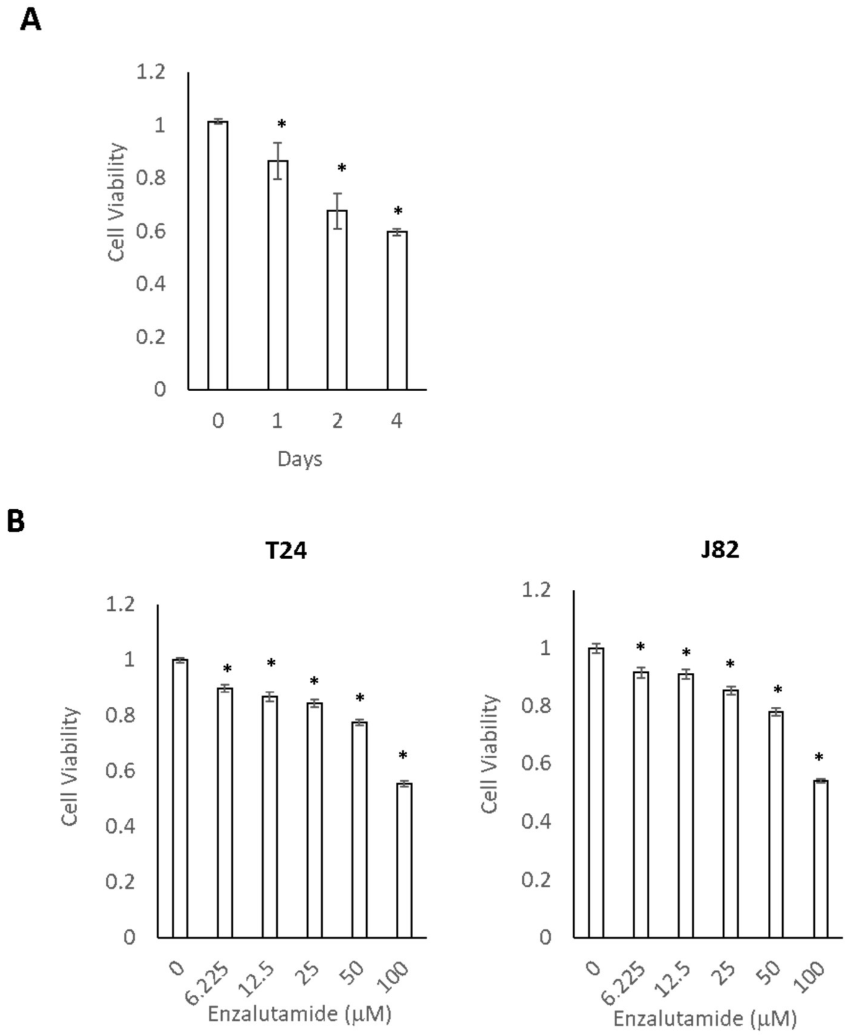

Figure S4. AR inhibition reduced cell viability of bladder cancer cells. (**A**) The relative cell viability after AR siRNA transfection to T24 cells divided with control siRNA treated cells. Bars represent the means ± SD of three independent experiments, and * denotes $p < 0.05$ (student *t*-test) versus the control siRNA group. (**B**) The relative cell viability after indicated concentrations of enzalutamide treatment to T24 or J82 cells. Bars represent the means ± SD of three independent experiments, and * denotes $p < 0.05$ (student *t*-test) versus the mock treated group.

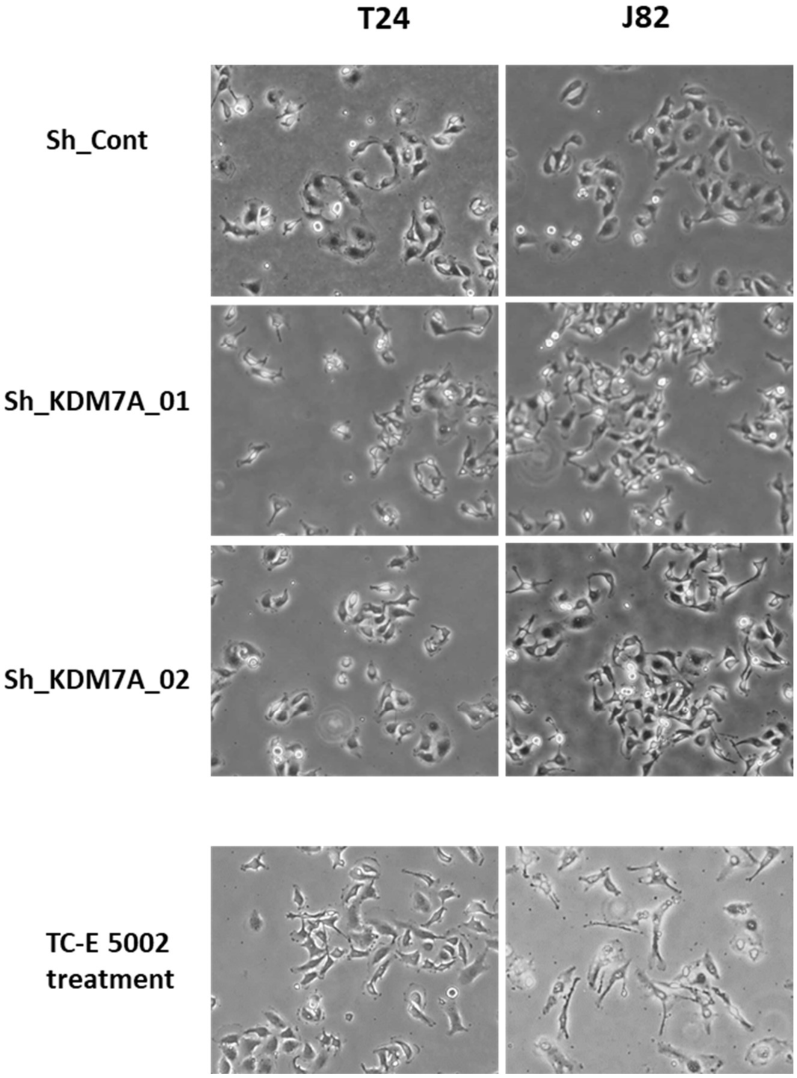

Figure S5. T24 and J82 bladder cancer cells expressing KDM7A shRNAs or treated with TC-E 5002 are photographed under phase contrast microscope.

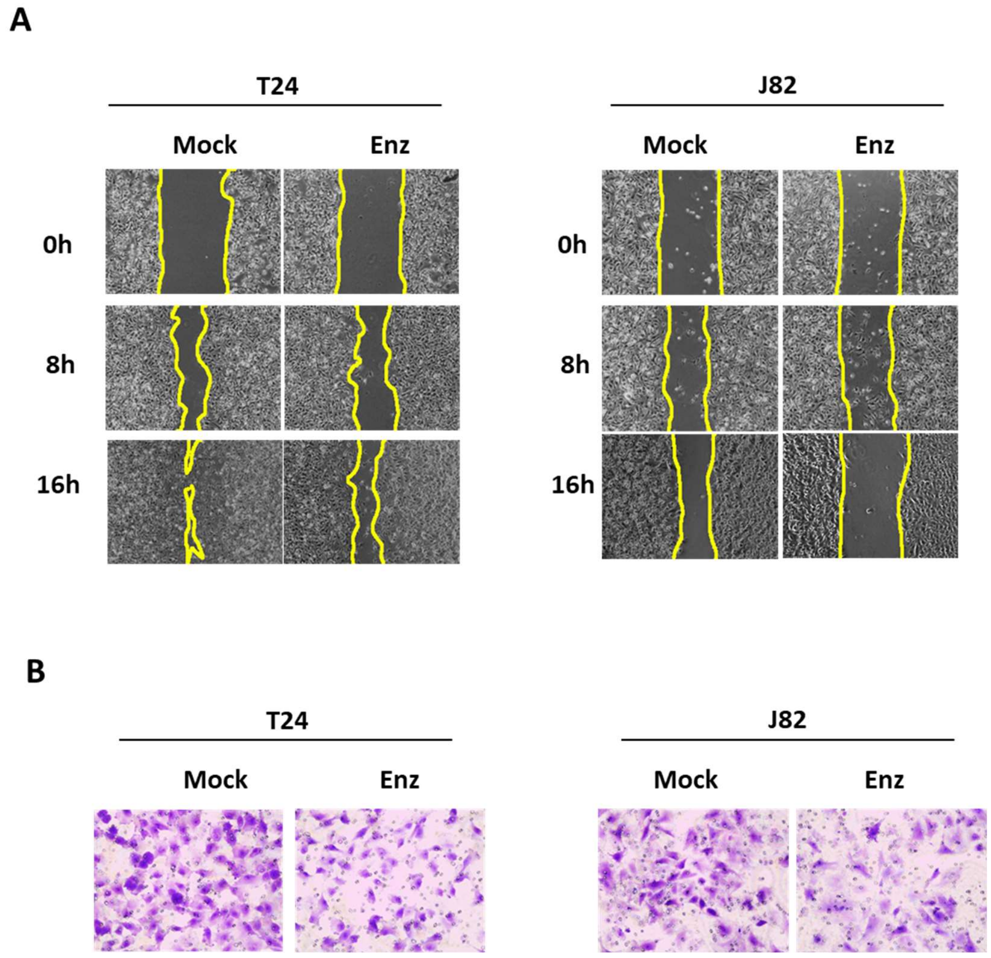

Figure S6. Enzalutamide treatment reduced cell mobility in bladder cancer cells. (**A**) Scratch-wounding cell migration assay of the indicated cells were observed for indicated time. (**B**) The Transwell assay of the same number of control and enzalutamide treated cells. At 24 h after plating, cells that had migrated to the underside of the filters were fixed and stained with crystal violet.

A

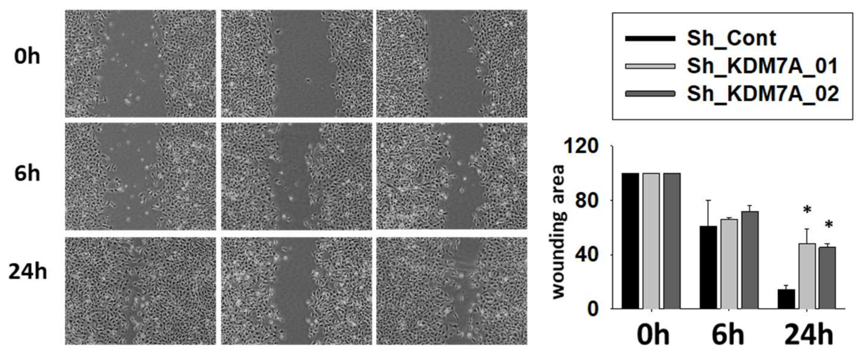

B

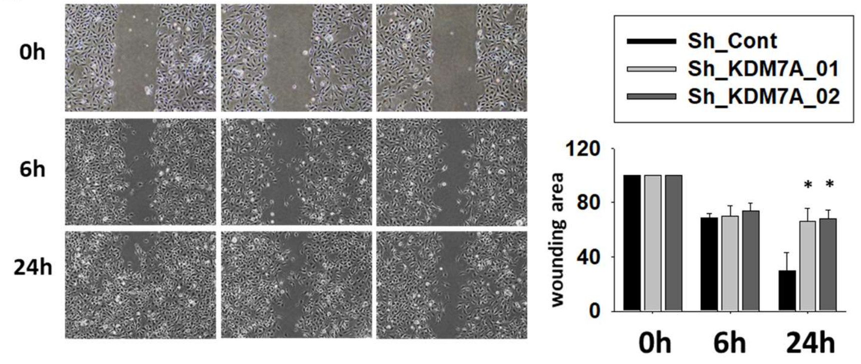

Figure S7. KDM7A knock-down reduced cell mobility in bladder cancer cells. Scratch-wounding cell migration assay of the control and KDM7A shRNA expressing T24 (**A**) and J82 (**B**) cells were observed for indicated time. Displayed pictures are the originals of the Figure 4A. The extent of scratched areas remained after indicated time are measured with Image J and plotted in right bar graphs. Bars represent the means ± SD of three independent experiments, and * denotes p < 0.05 (student t-test) versus the control shRNA (sh-cont) group.

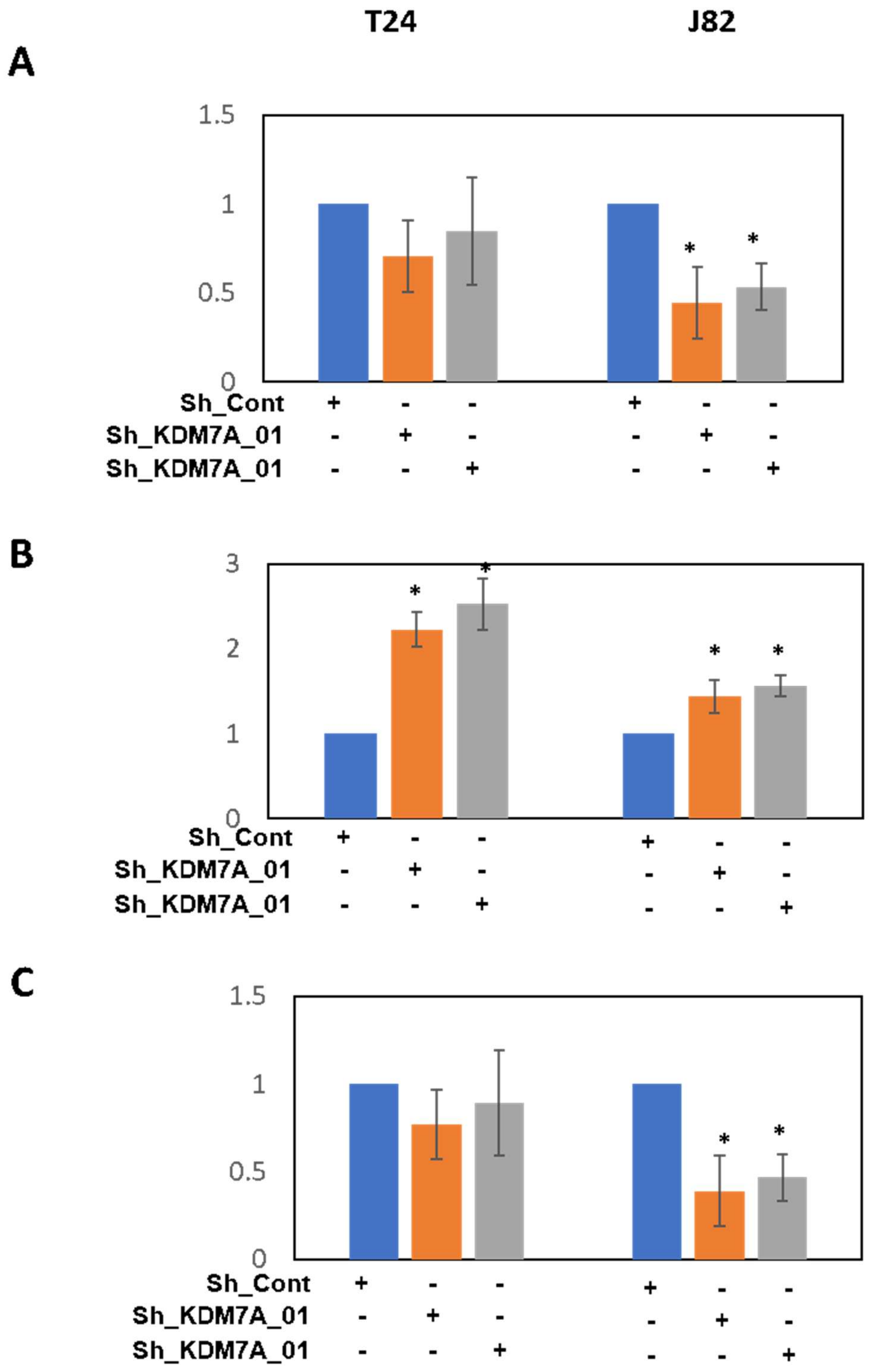

Figure S8. Protein bands from Figure 4C were analyzed densitometrically and protein levels were normalized to beta-actin levels. (**A**) E-CAD, (**B**) E-CAD, (**C**) Vimentin. Bars represent the means ± SD of three independent experiments. * $p < 0.05$ (Student's t-test) versus the sh_cont group.

A

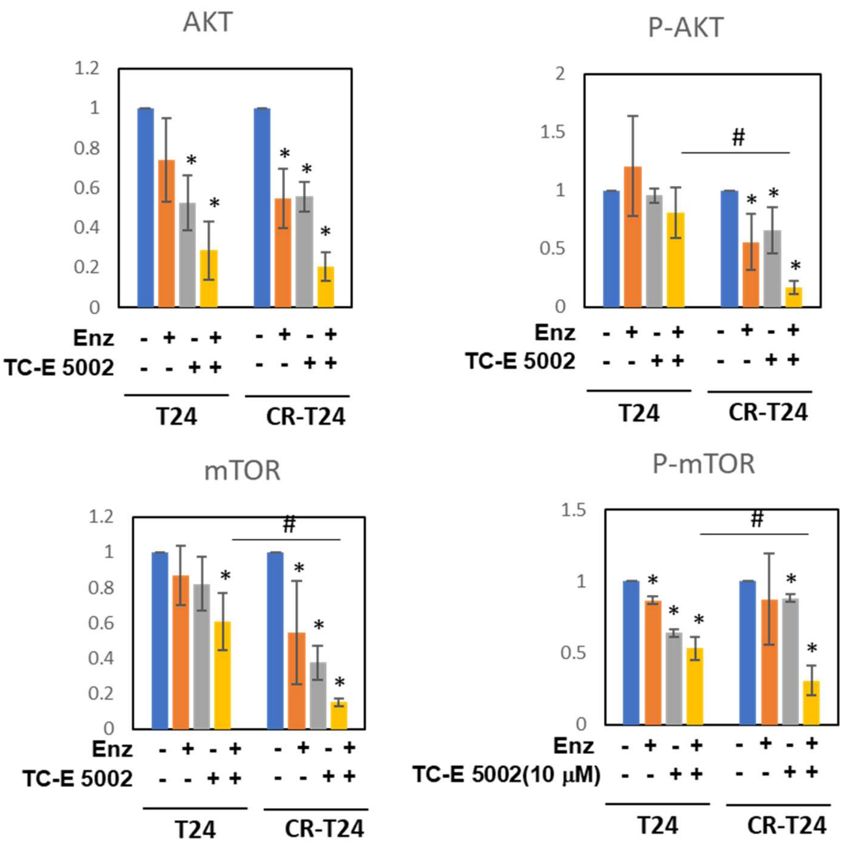

B

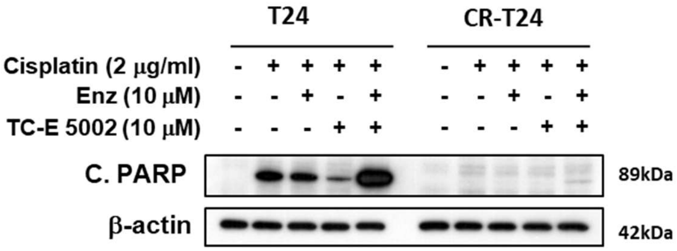

Figure S9. The enzalutamide and TC-E 5002 treatment reduces cell growth and increased apoptosis in cisplatin resistant T24 cells. (**A**) Protein bands from Figure 5F were analyzed densitometrically and protein levels were normalized to GAPDH levels. Bars represent the means ± SD of three independent experiments. * $p < 0.05$ (Student's *t*-test) versus the mock treated group. # $p < 0.05$ (Student's *t*-test) versus the parental T24 group. (**B**) The cleaved PARP protein level was analyzed after 2 days of indicated drugs.

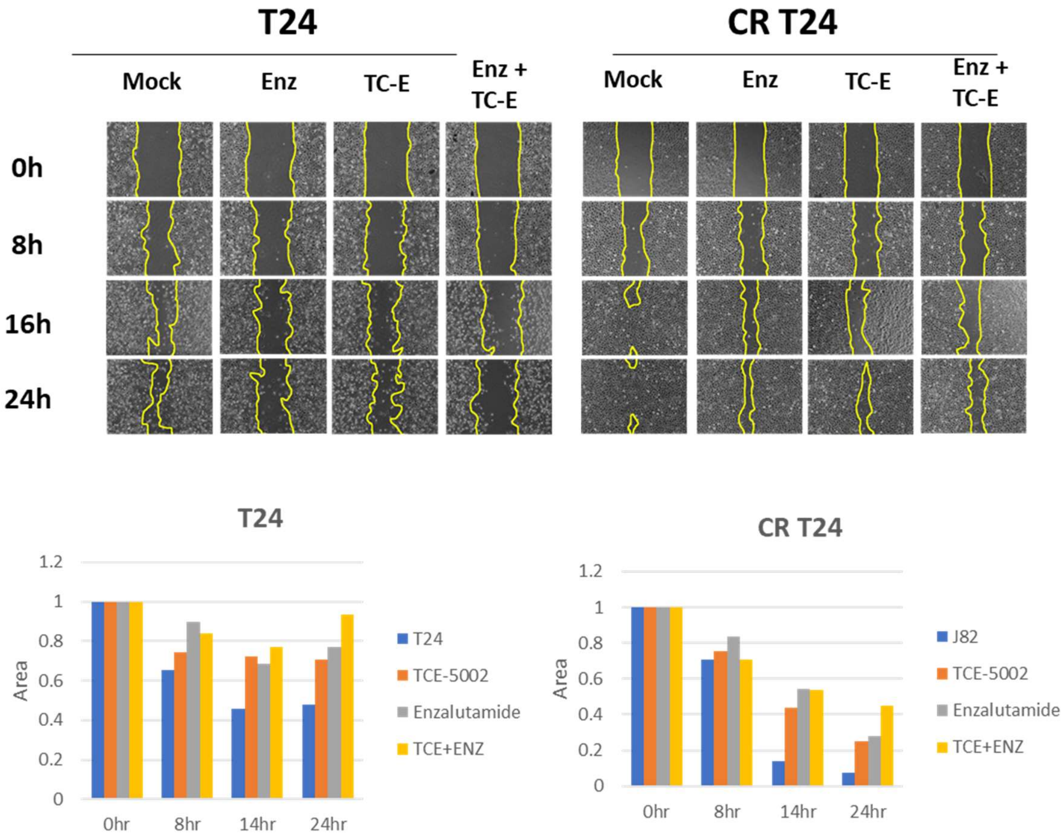

Figure S10. Enzalutamide and TC-E 5002 treatment reduced cell mobility in bladder cancer cells. Scratch-wounding cell migration assay of the parental and CR-T24 cells were observed for indicated time. The extent of scratched areas remained after indicated time are measured with Image J program and plotted in bar graphs under the pictures.

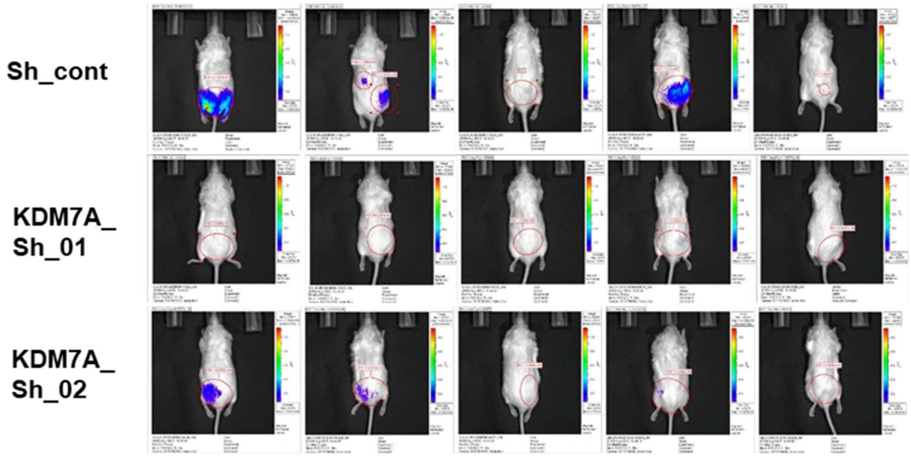

Figure S11. IVIS images demonstrating tumor formation on the day of sacrifice.

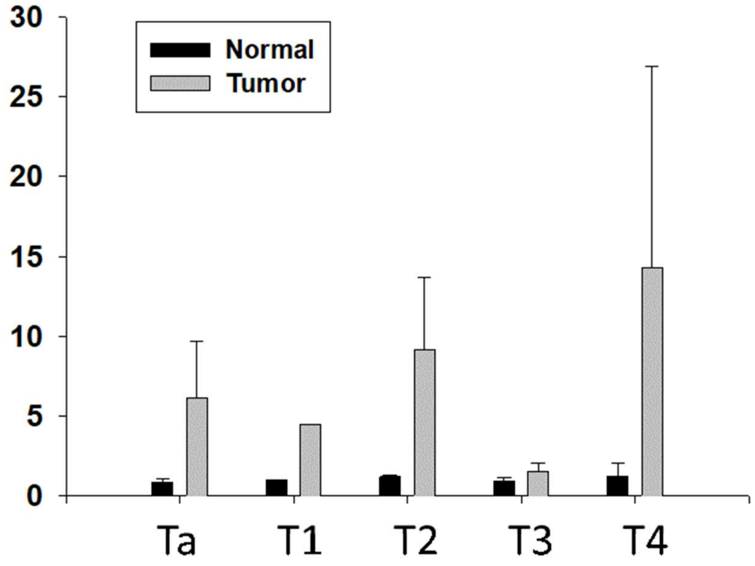

No.	Age	Sex	T stage	Normal	Tumor
1	66	M	T2bN0(0/21) LVI necrosis	1	1.75
2	73	F	T2aN0(0/16)	1.26	8.58
3	58	M	TaN0(0/25) CIS	1.06	2.65
4	56	F	T1N0(0/37)	1.05	4.47
5	84	M	T4aN0(0/1) LVI, Perineural invasion	0.48	1.73
6	67	M	TaN0(0/26)	0.71	9.70
7	64	M	T3aN2(2/7) LVI, Perineural invasion	0.83	0.52
8	75	M	T3bN1(1/17), Perineural invasion	0.67	1.62
9	72	M	T2aN0(0/14)	1.34	17.23
10	70	M	T3aN0(0/15) Lymphatic invasion	1.48	1.15
11	61	M	T4aN2(3/17), LVI Perineural invasion, necrosis	2.03	26.91
12	63	M	T3aN0(0/13)	0.79	2.89

Figure S12. KDM7A protein expression in human bladder tumor tissues and normal tissues from the same patient. The KDM7A protein band intensities from the western blots of Figure 8B were measured with Image J and compared with the tumor stages. Bars represent the means ± SD of each group. Patient demographic and calculated KDM7A intensities are listed in the table.

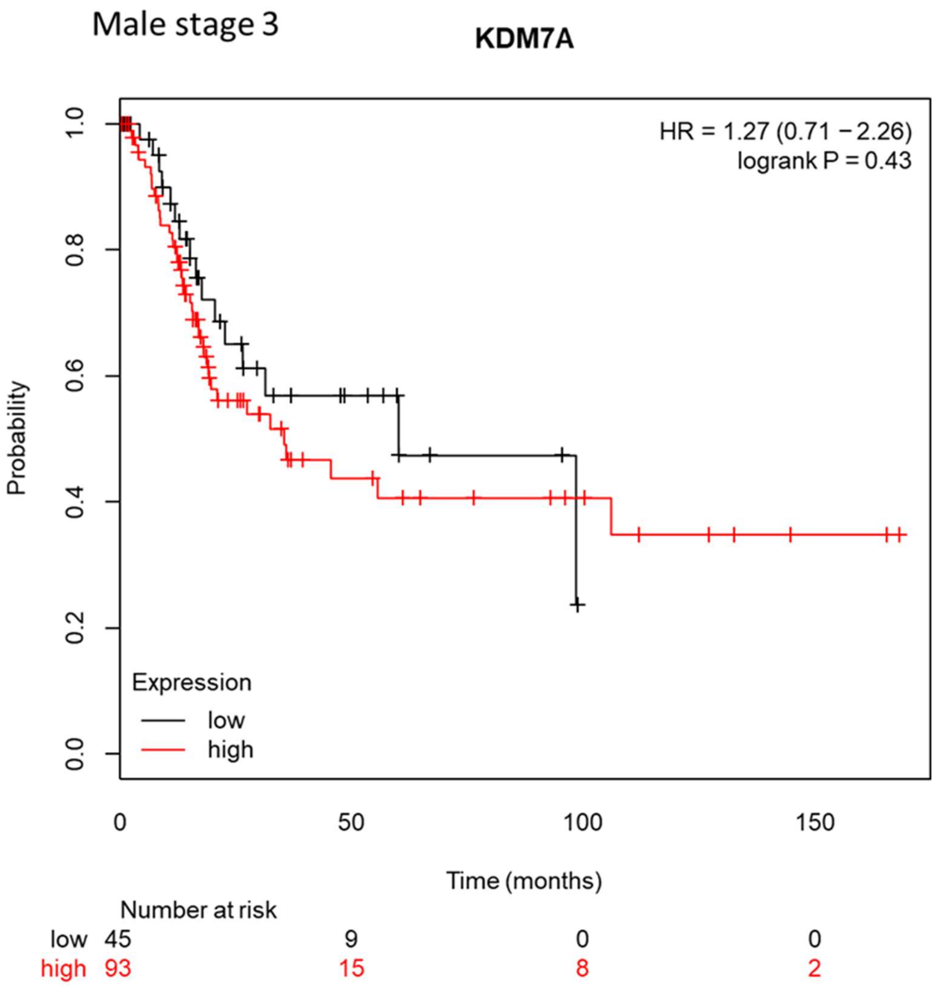

Figure S13. A survival curve was plotted for male bladder cancer patients with cancer stage 3 (n = 138). Data were analyzed from the Kaplan-Meier Plotter (www.kmplot.com). Patients with expression above the median are indicated in red line, and patients with expressions below the median in black line. HR means hazard ratio.

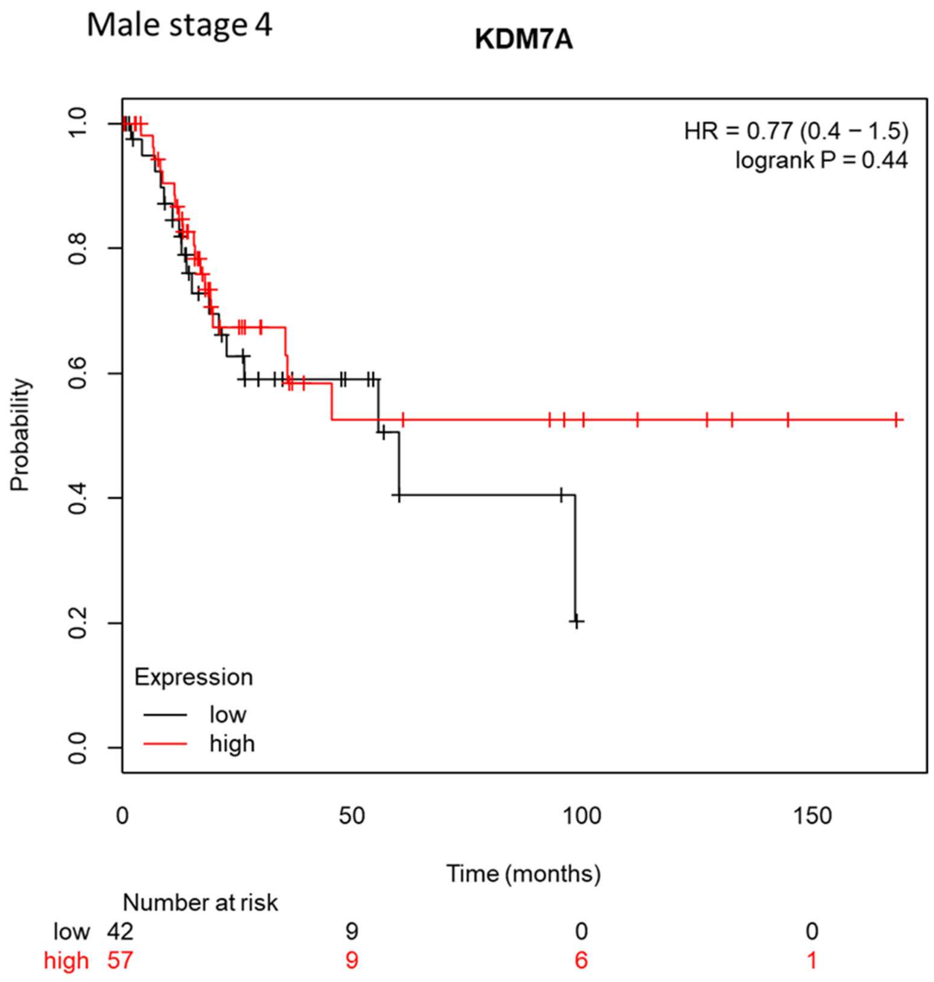

Figure S14. A survival curve was plotted for male bladder cancer patients with cancer stage 4 (n = 99). Data were analyzed from the Kaplan-Meier Plotter (www.kmplot.com). Patients with expression above the median are indicated in red line, and patients with expressions below the median in black line. HR means hazard ratio.

Table S1. The company and catalog numbers of antibodies.

Antibody Name	Company	Catalog No.
Anti-KDM7A	Novus (IHC,western)	NBP1-81282
Anti-KDM7A	Thermo Fisher (western)	PA5-25040
Anti-b-actin	Sigma-aldrich	A2066
Anti-N-cadherin	Cell Signaling	#13116
Anti-E-cadherin	Cell Signaling	#3195
Anti-vimentin	Abcam	ab92547
Anti-AR	Cell Signaling	#5153
Anti-Lamin B1	Santa Cruz Biotechnology	sc-6216
Anti-GAPDH	Santa Cruz Biotechnology	sc-32233
Anti-mTOR	Cell Signaling	#2983
Anti-P-mTOR	Cell Signaling	#5536
Anti-Akt	Cell Signaling	#9272
Anti-phospho-Akt	Cell Signaling	#9271
Anti-Ki-67	Abcam	ab92742
Anti-IGF1R	Santa Cruz Biotechnology	sc-712
Anti-TMPRSS2	Santa Cruz Biotechnology	sc-33533
Anti-VEGF	Santa Cruz Biotechnology	sc-7269
Anti-H3K4Me2	Cell Signaling	9725S
Anti-H3K9Me2	Cell Signaling	4658S
Anti-H3K27Me2	Cell Signaling	9728S
Anti-H3K36Me2	Cell Signaling	2901S
Anti-Histone H3	Cell Signaling	4499S

Table S2. Oligonucleotide sequences for RT-PCR and ChIP-PCR.

**Primer Sequences for RT-qPCR**	
18S_rRNA_RT_Fwd	TTCGTATTGAGCCGCTAGA
18S_rRNA_RT_Rev	CTTTCGCTCTGGTCCGTCTT
hAR_RT_Fwd	GGCGACAGAGGGAAAAAGG
hAR_RT_Rev	CCTTGCTTCCTCCGAGTCTT
hKDM7A_RT_Fwd	CCTTCACCCCACCAAGAGAC
hKDM7A_RT_Rev	AGACGTTGTTTGGCTGTTGC
hKLK3_RT_Fwd	CACCTGCTCGGGTGATTCTG
hKLK3_RT_Rev	CCACTTCCGGTAATGCACCA
hKLK4_RT_Fwd	AGGATCGCTCGTCTCTGGTA
hKLK4_RT_Rev	GGAGCTCTGCACTCACTTCT
hTMPRSS2_RT_Fwd	GGACAGTGTGCACCTCAAAGA
hTMPRSS2_RT_Rev	TTGCTGCCCATGAACTTCC
hVEGF_RT_Fwd	TGCATTCACATTTGTTGTGC
hVEGF_RT_Rev	AGACCCTGGTGGACATCTTC
hIGF1R_RT_Fwd	GGGCCATCAGGATTGAGAAA
hIGF1R_RT_Rev	CACAGGCCGTGTCGTTGTCA
hMYC_RT_Fwd	TACAACACCCGAGCAAGGAC
hMYC_RT_Rev	TTCTCCTCCTCGTCGCAGTA
hECAD_RT_Fwd	TCGGACCAAGGACAAGTACC
hECAD_RT_Rev	ATCTTCACCTGCCGTTCAGT
hNCAD_RT_Fwd	GACAATGCCCCTCAAGTGTT
hNCAD_RT_Rev	CCATTAAGCCGAGTGATGGT
hVIM_RT_Fwd	GAGAACTTTGCCGTTGAAGC
hVIM_RT_Rev	GCTTTCTGTAGGTGGCAATC
**Primer sequences for ChIP-PCR**	
hKLK3 _ChIP_Fwd	GGGATCAGGGAGTCTCACAA
hKLK3 _ChIP_Rev	GCTAGCACTTGCTGTTCTGC
hKLK2_ChIP_Fwd	GCCTTCTCTGGCTTTGTTCC
hKLK2_ChIP_Rev	GCACTTGCTGTTCCACACAT
hTMPRSS2_ChIP_Fwd	TGGTCCTGGATGATAAAAAAAGTTT
hTMPRSS2_ChIP_Rev	GACATACGCCCCACAACAGA
hVEGF_ChIP_Fwd	TTCGAGAGTGAGGACGTGTG
hVEGF_ChIP_Rev	AGGGAGCAGGAAAGTGAGGT
